# RNA transcription and degradation of Alu retrotransposons depends on sequence features and evolutionary history

**DOI:** 10.1093/g3journal/jkac054

**Published:** 2022-03-07

**Authors:** Till Baar, Sebastian Dümcke, Saskia Gressel, Björn Schwalb, Alexander Dilthey, Patrick Cramer, Achim Tresch

**Affiliations:** 1 Institute of Medical Statistics and Computational Biology, Faculty of Medicine, University of Cologne, Cologne 50937, Germany; 2 Clemedi AG, Schlieren 8952, Switzerland; 3 Department of Molecular Biology, Max Planck Institute for Biophysical Chemistry, Göttingen 37077, Germany; 4 Institute of Medical Microbiology and Hospital Hygiene, Medical Faculty, Heinrich-Heine-University Düsseldorf, Düsseldorf 40225, Germany; 5 CECAD, University of Cologne, Cologne 50931, Germany; 6 Center for Data and Simulation Science, University of Cologne, Cologne 50923, Germany

**Keywords:** Alu elements, Alu transcription, RNA labeling, retrotransposons

## Abstract

Alu elements are one of the most successful groups of RNA retrotransposons and make up 11% of the human genome with over 1 million individual loci. They are linked to genetic defects, increases in sequence diversity, and influence transcriptional activity. Still, their RNA metabolism is poorly understood yet. It is even unclear whether Alu elements are mostly transcribed by RNA Polymerase II or III. We have conducted a transcription shutoff experiment by α-amanitin and metabolic RNA labeling by 4-thiouridine combined with RNA fragmentation (TT-seq) and RNA-seq to shed further light on the origin and life cycle of Alu transcripts. We find that Alu RNAs are more stable than previously thought and seem to originate in part from RNA Polymerase II activity, as previous reports suggest. Their expression however seems to be independent of the transcriptional activity of adjacent genes. Furthermore, we have developed a novel statistical test for detecting the expression of quantitative trait loci in Alu elements that relies on the de Bruijn graph representation of all Alu sequences. It controls for both statistical significance and biological relevance using a tuned *k*-mer representation, discovering influential sequence features missed by regular motif search. In addition, we discover several point mutations using a generalized linear model, and motifs of interest, which also match transcription factor-binding motifs.

## Introduction

Alu elements are around 300-bp-long RNA retrotransposons (Lander *et al.* 2001, for review, see Deininger *et al.* 2011). They are classified as short interspersed nuclear elements (SINEs) and are highly abundant in the genome of higher primates (Gentles *et al.* 2005). Over 1 million Alu loci are currently annotated in the human genome. This means that 11% of the whole genome consists of just Alu sequences (Lander *et al.* 2001), making them one of the most successful groups of mobile elements.

Alu elements were discovered using a restriction endonuclease of *Athrobacter luteus*, which gave them their name (Schmid and Deininger 1975). They are RNA retrotransposons, also called class I transposable elements, and as such are capable of copying themselves into new positions in the genome.

**Fig. 1.**
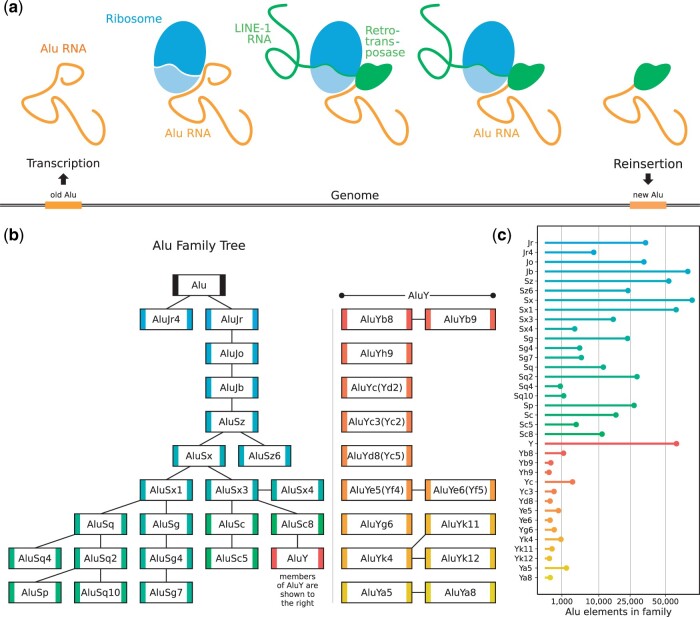
Alu evolution and retrotransposition. a) Schematic representation of the Alu element reinsertion process (left to right). The Alu element is transcribed. The Alu RNA attaches itself to the exit tunnel of the ribosome through its SRP sequence homolog. A LINE-1 RNA arrives at the ribosome and its retrotransposase is translated. The Alu RNA hijacks the LINE-1 retrotransposase. The LINE-1 retrotransposase reinserts the Alu element into the genome at a new position. b) Human Alu family tree without designation of approximate evolutionary age (Price *et al.* 2004). Members of AluY are shown to the right. Alternative family naming conventions are denoted in brackets. The color scheme is reused in subsequent figures. c) Individual Alu Elements per family in the human genome (UCSC Genome Browser Annotation).

As shown schematically in Fig. 1a, once an Alu element is transcribed, its RNA attaches itself to the exit tunnel of the ribosome, using a sequence homolog to the signal recognition particle (SRP), a ribonucleoprotein part of the eukaryotic ribosome (Conti *et al.* 2015; Tisdale and Pellizzoni 2017). As Alu elements are nonautonomous retrotransposons, they lack their own retrotransposase domain. Instead, they rely on autonomous long interspersed nuclear elements (LINE)-1 elements, another class of retrotransposon belonging to the LINEs that cover up to 17% of the genome (Boeke 1997; Dewannieux *et al.* 2003). Thus, the Alu RNA lies in wait at the ribosome’s exit tunnel until a LINE-1 element is translated. It then uses the LINE-1 retrotransposase to reinsert itself into a new position in the genome (Häsler and Strub 2006).

The Alu life cycle carries with it certain risks for the host organism, as Alu element insertion into genes or other functional genomic regions can disrupt them (Deininger and Batzer 1999). As such, Alus have also been linked to increases in sequence diversity (Kazazian 2004; Ade *et al.* 2013), as well as influencing transcriptional activity in general (Chen and Yang 2017; Zhang *et al.* 2019), and under heat shock conditions, in particular (Mariner *et al.* 2008). While generally lowly abundant, Alu elements are expressed and do successfully reinsert themselves into the human genome, with an estimated new Alu insertion for every 20 children born (Han *et al.* 2007; Xing *et al.* 2009; Conti *et al.* 2015).

While the exact evolutionary origin of Alu elements is unknown, they are assumed to be derived from the 7SL RNA, which is itself a component of the SRP (Ullu and Tschudi 1984; Kriegs *et al.* 2007). They possess a dimeric structure with a left arm and a right arm, separated by a variable A-rich region, as is also shown later in Fig. 5 (Evgen’ev 2007). In addition, they possess 2 sequence features of note, the first being a bipartite RNA polymerase III (Pol-III) promoter located in the left arm, which is split into A box and B box (Paolella *et al.* 1983; Orioli *et al.* 2012). The second is the UGU(NR)-binding motif required for attachment to the ribosome exit tunnel (DagAn *et al.* 2004).

While only present in higher primates, Alu elements most likely evolved from B1 repeats in rodents, which became the free left and right Alu monomers (FLAM and FRAM) in primates, which lastly fused to form the Alu elements (Quentin 1992; Kriegs *et al.* 2007). However, Alu evolution through error-prone retrotransposition continues and has given rise to many Alu families (Richard Shen *et al.* 1991; Deininger *et al.* 1992). The evolutionary oldest is AluJ, followed by AluS, and finally, AluY, which shows the most transposition activity in humans (Batzer *et al.* 1996). An Alu family tree is shown in Fig. 1b (.json encoding in Supplementary material S1).

Despite the presence of a Pol-III promoter in their sequence, Alu elements are expected to be transcribed not only by Pol-III but also by RNA polymerase II (Pol-II) (Conti *et al.* 2015; Zhang *et al.* 2019). In this study, we investigate different hypotheses regarding Alu transcript origins further, using metabolic RNA labeling by 4-thiouridine [dynamic transcriptome analysis (DTA), Schwalb *et al.* 2012] coupled with the inhibition of Pol-II (see *Methods*, *Alu RNA* used interchangeably with *Alu transcripts*). This is of particular interest regarding past experiments that utilized Pol-II inhibition, as it is common practice to rely on SINEs as a non-Pol-II-dependent negative control group in such cases.

We also explore the half-life of Alu RNA in the cell, which is expected to be low, as the transcripts are presumed to be unstable (An *et al.* 2004). Finally, we also analyze the totality of annotated Alu loci to detect sequence features that influence Alu transcription. While standard motif search has been applied to this problem with limited success in previous studies (Zhang *et al.* 2019), we use a base-level generalized linear model (Nelder and Wedderburn 1972), and alternatively a colored and compacted de Bruijn graph (Idury and Waterman 1995; Pevzner *et al.* 2001; Iqbal *et al.* 2012), which lead to the de novo discovery of highly significant motifs that can partly be assigned to known transcription factors.

## Methods

### Data sets

The sequencing data used in this investigation are described in detail in our previous publication Schwalb *et al.* (2016), exception for the sequencing data relating to the inhibition of RNA Pol-II by α-amanitin, which was newly generated. Both data sets are available in NCBI’s Gene Expression Omnibus, as described in *Data Availability*, including a table containing the positions, counts, and differential expression analysis results for the examined RNAs. The data set from Schwalb *et al.* (2016) is used throughout this investigation, except where α-amanitin inhibition is concerned.

### K562 cells

Human K562 erythroleukemia cells were obtained from DSMZ (Cat. # ACC-10; RRID: CVCL_0004). K562 cells were cultured in accordance with the DSMZ Cell Culture standards in RPMI 1640 medium (Thermo Fisher Scientific) containing 10% heat inactivated fetal bovine serum (Thermo Fisher Scientific), 1% penicillin–streptomycin, and 1× GlutaMAX supplement (Thermo Fisher Scientific) at 37°C in a humidified 5% CO_2_ incubator. K562 cells used in this study display the phenotypic properties, including morphology and proliferation rate, that have been described in the literature. Cells were verified to be free of mycoplasma contamination using the Plasmo Test Mycoplasma Detection Kit (InvivoGen). Biological replicates were cultured independently.

### α-Amanitin treatment

α-Amanitin is a toxic substance from the mushroom *Amanita phalloides* and a potent inhibitor of RNA Pol-II (Lindell *et al.* 1970; Kedinger *et al.* 1970; Stirpe and Fiume 1967; Jacob *et al.* 1970). Among the RNA polymerases, RNA Pol-II is inhibited at low α-amanitin concentrations, RNA Pol-III may be inhibited at high concentrations (>250 µg mL^−1^), while RNA polymerase I (Pol-I) and mitochondrial RNA polymerases remain unaffected by α-amanitin. Treatment conditions were optimized for selective RNA Pol-II inhibition (see RT-qPCR and Western blotting). For α-amanitin (5 or 15 µg mL^−1^) treatments of K562 cells, cells were treated for a time course of 0–9 h at 37°C in a humidified 5% CO_2_ incubator. For TT-seq and RNA-seq, K562 cells were treated for 8 h at 37°C in a humidified 5% CO_2_ incubator with 5 µg mL^−1^ α-amanitin or solvent (water).

### RT-qPCR

For each condition (5 or 15 µg mL^−1^ α-amanitin or solvent), $5\times 10^{6}$ cells were harvested at 3,000 × *g* for 2 min. Total RNA was isolated with QIAzol (QIAGEN) according to manufacturer’s instructions except for the addition of 10 ng RNA spike-in mix (6 spike-ins selected from ERCC RNA spike-in mix) (Schwalb *et al.* 2016) together with QIAzol. To remove possible genomic DNA contamination, isolated RNA (10 ng) was treated with TURBO DNase (Thermo Fisher Scientific) according to manufacturer’s instructions. For reverse transcription (RT), random hexamer priming [5′-d(NNNNNN)-3′, N = G, A, T, or C] was used according to manufacturer’s instructions. Briefly, 1 µg of DNase-treated RNA, Random Hexamer primers (final concentration of 5 ng µL^−1^), and dNTP mix (final concentration of 0.5 mM) were mixed and incubated at 65°C for 5 min. Subsequently, Maxima H Minus Reverse Transcriptase (RT) (final concentration of 200 U) and 5× Maxima RT buffer (Thermo Fisher Scientific) were added (+RT reaction). For DNA contamination control, cDNA synthesis without RT (−RT reaction) was performed (RT was substituted with water). The (−/+) RT reactions were incubated in a PCR cycler at 25°C for 10 min, 50°C for 30 min, and 85°C for 5 min. Primers for quantitative PCR were designed by using the online primer design software Primer3 v.0.4.0 (Rozen and Skaletsky 2000). Briefly, the selection of Pol-II targets was based on half-lives measurements in K562 cells (Schwalb *et al.* 2016). Primers were targeted to Pol-II transcripts (PAIP1, CWC22, EGR1) as a positive control, RNA Pol-I transcripts (18S rRNA) as a negative control, and RNA Pol-III transcripts (U6 snRNA). Primer specificity (single product peak) was validated by melting profiles. Primer sequences, length, PCR efficiency values of primers (E) and targets are reported in Supplementary material S2. cDNAs (50 ng) were amplified with SYBR Select Master Mix (Thermo Fisher Scientific) according to the manufacturer’s instruction with a final primer concentration of 400 nM. PCRs were run in 96-well optical plates sealed with optical adhesive cover on a qTOWER 2.0/2.2 instrument (Analytik Jena AG). The following thermal cycling conditions were used (SYBR Select Master Mix reference, standard cycling mode): 50°C for 2 min, 95°C for 2 min, 40 cycles of 95°C for 15 s, and 60°C for 1 min. Two synthetic RNA spike-ins were used for normalization. The $2^{-\Delta \Delta \;\text{Ct}}$ method was applied to calculate the normalized target gene expression fold change, with the amplification efficiency (*E*) for each target gene, slope of standard curve (*S*), and mean threshold cycle (Ct) (Livak and Schmittgen 2001).

### Western blot

For α-amanitin (5 or 15 µg mL^−1^) treatments of K562 cells, cells were treated for 8 h at 37°C in a humidified 5% CO_2_ incubator. Water was used as a solvent. After 8 h of treatment, K562 cells were harvested at 3,000 × *g* for 2 min and washed twice in Dulbecco's phosphate-buffered saline (DPBS). Cells were lysed in radioimmunoprecipitation assay (RIPA) lysis buffer for 45 min on ice and centrifuged at 14,000 × *g* for 15 min at 4°C. Samples were quantified with the Bradford method. Fifteen micrograms of cell lysate was denatured in 4× Loading Dye (including 100 mM DTT) at 70°C for 10 min. PrecisionPlus Protein All Blue Standard (Bio-Rad, # 161-0373, 10–250 kDa) was used as marker. NuPAGE 4–12% Bis–Tris Protein Gels and MOPS buffer were used according to manufacturer’s instructions. Transfer of a single NuPAGE onto PVDF membrane was performed in transfer buffer at 30 V for 1 h in a XCell II Blot Module (semiwet transfer unit) according to manufacturer’s instructions. Membrane blocking was performed in 5% milk PBS-T on a rocking surface for at least 1 h. Primary antibody was added overnight. The following primary antibodies were used in this study: N-terminal POLR2A/hRPB1 antibody, clone F-12 (Santa Cruz Biotechnology, sc-55492, Lot. # E2913; RRID: AB_630203) and GAPDH antibody, clone C 71.1 (Sigma-Aldrich, G8795, Lot. # 067M4785V; RRID: AB_1078991) as a loading control. HRP-coupled secondary antibody targeting mouse IgG (Abcam, ab5870) was used at a dilution of 1:3,000 and incubated on a rocking surface for 1 h. Enhanced chemiluminescenc (ECL) working solution of SuperSignal West Pico PLUS Chemiluminescent Substrate was prepared according to manufacturer’s instructions. Proteins were visualized by chemiluminescence detection on INTAS. POLR2A/hRPB1 (RNA Pol-II) degradation, which is α-amanitin dose dependent (Nguyen *et al.* 1996), was monitored by Western blotting.

### RNA spike-ins

Synthetic RNA spike-in controls are derived from selected RNAs of the ERCC RNA Spike-in Mix (Ambion) as described in Gressel *et al.* (2019b) and Schwalb *et al.* (2016). Briefly, spike-ins (3 unlabeled and 3 4sU labeled) are in vitro transcribed using the MEGAscript T7 kit (Ambion). In vitro transcription (IVT) of unlabeled spike-ins was performed following the manufacturer’s instruction. For IVT of 4sU-labeled spike-ins, 10% of UTP was substituted with 4-thio-UTP (Jena Bioscience). RNA spike-ins were purified with RNAClean XP beads (Beckman Coulter) following manufacturer’s instructions. The final RNA spike-in pool contained equal amounts of all RNA spike-ins.

### TT-seq and RNA-seq

A detailed step-by-step protocol has been deposited in the protocols.io repository (Gressel *et al.* 2019a). TT-seq and RNA-seq were performed in 2 biological replicates including RNA spike-ins. Briefly, experiments were performed using $5\times 10^{7}$ K562 cells per biological replicate. Cells were kept at optimal growth conditions and supplemented with 5 µg mL^−1^ of α-amanitin or solvent (water) for 8 h. After 7 h 55 min, a 4-thiouridine (4sU) labeling pulse (Sigma-Aldrich, T4509) was applied for 5 min using 500 µM (see Supplementary material S3). Total RNA was isolated with the QIAzol reagent (# 79306) according to manufacturer’s instructions except for the addition of 150 ng of RNA spike-in pool with QIAzol reagent as previously described (Schwalb *et al.* 2016; Gressel *et al.* 2019a). The Ovation Universal RNA-Seq System (NuGEN) was used for strand-specific library preparation as described (Gressel *et al.* 2019b). Purified cDNA libraries were analyzed by Fragment Analyzer prior to Illumina sequencing. Sequencing was performed on a HiSeq 2500 (Illumina) in paired-end mode with 50-bp read length.

### TT-seq/RNA-seq prepossessing and normalization parameters

TT-seq and RNA-seq prepossessing and normalization were performed as detailed in Schwalb *et al.* (2016), with an alternative normalization applied as described under *Differential Expression Analysis*. Reads were mapped with STAR v2.7.3a (Dobin *et al.* 2016), allowing a maximum of 10 mismatches and minimal uniqueness filtering with an MAPQ value cutoff of 255. The general error rate per library ranged between 0.6% and 0.9%. As Alu sequences are notoriously difficult to map (Sexton and Han 2019), we performed a mappability analysis (see Supplementary material S4) to make certain that our read alignment does not introduce biases. We simulated a homogeneous coverage of the genome and mapped the simulated reads using the same aligner settings. Our analysis shows that there is no bias caused by Alu mappability, which is also corroborated by the findings of Sexton and Han (2019), who show that the mappability of transposable elements can be improved through the use of paired-end read libraries to the point where a majority of elements are uniquely mappable. Association of the sequencing data with specific Alu elements or protein-coding genes used the GRCh37/hg19 repeat annotation or respectively the canonical gene annotation of the UCSC Table Browser (Karolchik *et al.* 2004), in addition to the RepeatMasker human Alu subfamily re-analysis (Price *et al.* 2004). We also tested using the transcription unit annotation from Gressel *et al.* (2019b) based on the GenoSTAN segmentation algorithm as an alternative to the UCSC canonical gene annotation, which leads to a similar mRNA read count distribution (Zacher *et al.* 2017, data not shown).

### RNA half-life estimation

To estimate the half-life of expressed Alu elements and coding transcripts, we used MLE, described in detail in Supplementary material S5. Our estimation makes use of the labeled and total RNA sequencing fractions obtained through TT-seq and 4sU-seq, as detailed above. TT-seq and 4sU-seq data were used for the estimation, as the correlation between the 2 methods is suitably strong (r>0.80 between replicates and r>0.85 between sequencing methods). While 4sU-seq was designed to estimate half-lives, TT-seq was primarily designed to measure the polymerase processivity during transcription. Since Alu elements are short, TT-seq essentially measures the synthesis rate of Alu transcripts and can thus be applied for half-life estimation (see Supplementary material S6).

We assume that the total amount of transcripts in a cell remains constant and that transcript degradation follows exponential decay, meaning that the ratio of labeled to total transcripts *L*/*T* increases exponentially to 1 over time. We further assume that the distribution of labeled and total counts follow a Poisson distribution. This allows us to formulate the likelihood function L of observing a specific number of labeled counts *L_a_* and total counts *T_a_* for any given Alu element or coding transcript *a*, as a parametrized combination of the underlying Poisson distribution:

$$\begin{array}{c} L(L_{a},T_{a};r_{a},q,L_{\mathrm{spk}},T_{\mathrm{spk}})=\text{Pois}(T_{a};\lambda_{\mathrm{tot}}=c_{\mathrm{tot}}\cdot r_{a}^{-1})\cdot \\ \text{Pois}(L_{a};\lambda_{\mathrm{lab}}=c_{\mathrm{lab}}\cdot r_{a}^{-1}) \end{array}$$

Here, *r_a_* is the ratio between labeled and total molecules for any given Alu element or coding transcript *a*; *q* is the ratio between labeled and total spike-in molecules that were added; *L_spk_* and *T_spk_* are the number of labeled and total spike-in counts respectively; and *c_lab_* and *c_tot_* are given as:

$$c_{\mathrm{tot}}:=\frac{L_{a}}{L_{\mathrm{spk}}}\cdot T_{\mathrm{spk}}\cdot q\;\text{and}\;c_{\mathrm{lab}}:=L_{\mathrm{spk}}\cdot \frac{T_{a}}{T_{\mathrm{spk}}}\cdot \frac{1}{q}$$

This estimation does not depend on feature length, as we use the ratio between labeled and total counts. To find the optimal estimate of *r_a_*, we maximize the log likelihood function:

$$\begin{array}{c} \ell (r_{a};L_{a},T_{a},q,L_{\mathrm{spk}},T_{\mathrm{spk}})=L_{a}\left(\ln\left(r_{a}\right)-\frac{T_{\mathrm{spk}}}{L_{\mathrm{spk}}}\frac{q}{r_{a}}\right)\\ +T_{a}\left(-\frac{L_{\mathrm{spk}}}{T_{\mathrm{spk}}}\frac{r_{a}}{q}-\ln\left(r_{a}\right)\right) \end{array}$$

From *r_a_* when then obtain the degradation rate *δ_a_* by use of $r_{a}=1-\text{exp}(-\delta_{a}\Delta t)$, where Δt is the labeling pulse’s duration. This allows us to calculate the half-life as $t_{1/2}=\ln\left(2\right)/\delta_{a}$.

As the used DTA methods rely on the labeling of U as 4sU, the percentage of U in an Alu sequence could potentially bias its half-life estimate. However, we can exclude this possibility, as no correlation exists between Alu half-life and U ratio (r<0.01).

### Differential expression analysis

Differential expression analysis of the α-amanitin Pol-II inhibition experiment was performed using the DESeq2 package for R (Love *et al.* 2014), using un-normalized counts as required by the statistical model. The standard normalization strategy employed by DESeq2 internally relies on the assumption that there are no substantial, systematic global changes in (mRNA) expression between samples. Since this is most likely not given in the α-amanitin sample, we used a set of *bona fide* housekeeping transcripts. As it is known that the activity of mitochondrial polymerase is unaffected by α-amanitin (Menon 1971; Reid and Parsons 1971; Saccone *et al.* 1971), we chose mtRNAs for normalization. Alternatively, to check the robustness of our choice, we also performed normalization based on a set of spike-in RNAs that had been added during sequencing library preparation (see *RNA Spike-Ins*). Similar results were obtained when spike-in RNAs were taken as reference as compared to mtRNAs (Supplementary material S7c). The normalization constants obtained differ by a mean factor of 1.10, and consequently the results of our analysis hold under both regiments.

Rejection sampling was used to ensure that the obtained l2fc distributions are comparable (Flury 1990). To avoid biases arising from the substantially different expression level distribution of Alus and mRNAs, we compare the Alu l2fc distribution to the l2fc distribution of a large sample of mRNAs that were picked randomly such that their wild-type expression distribution is identical to that of the Alu elements. To generate this random sample, we use rejection sampling: we define expression breakpoints

$$b_{0}=-\text{inf}<b_{1}<b_{2}<\cdots <b_{N-1}<b_{N}=\text{inf}$$

such that each bin $\left[b_{n},b_{n+1}\right]$ contains 1,500 Alu elements, excluding Alu elements with less than 5 reads. Thus, the empirical expression distribution

$$f\left(n\right)=p\left(\text{Alu expression in}\left[b_{n-1},b_{n}\right]\right)=1/N,\;n=1,\ldots ,N$$

is uniform. The same breakpoints are used to calculate the empirical expression distribution

$$g\left(n\right)=p\left(\text{mRNA expression in}\left[b_{n-1},b_{n}\right]\right),\;n=1,\ldots ,N$$

of the mRNAs by calculating the relative abundance of mRNAs whose expression falls into the interval $\left[b_{n-1},b_{n}\right]$. Let k=min(g​(n),n=1…,N) and accept an mRNA sample that lies in the interval *m* with probability k/g​(k). It can then be shown that the set of accepted samples approaches, for large numbers, the distribution f​(n).

### Generalized linear model

The generalized linear models (GLMs) to analyze the genomic sequences of all annotated Alu elements on base level were created with the glmnet package for R (Friedman *et al.* 2010). A Poisson family distribution response type was assumed with observation standardization, an elastic mixing parameter α of 1 (full Lasso penalty, no ridge regression penalty), no fitted intercept parameter, and 1,000× cross-validation. The input matrix for each model consisted of a binary encoding of the examined point mutation, base exchange, deletion, or insertion, for each position in the Alu consensus sequence. These matrices were obtained by aligning each individual Alu sequence against the Alu consensus sequence. These predictor matrices where then paired with the expression values as response variable for each individual model. The resulting effect sizes (β parameters) for the point mutations were then annotated along the Alu secondary structure according to Sinnett *et al.* (1991) (dot-bracket notation of the secondary structure in Supplementary material S8) to illustrate their context regarding Alu sequence features. The β parameters are the estimated regression coefficients of the GLM.

### de Bruijn graph

The de Bruijn graph of all Alu sequences, regardless of expression, was generated using bifrost v1.0.5 (Holley and Melsted 2020), with a *k*-mer length of 9 and including 100 bp of flanking regions up- and downstream of each Alu sequence. *k* = 9 was chosen so that the distribution of Alu sequences per node in the graph was smooth, neither running into the lower nor the upper range of possible values (Supplementary material S9). Low-coverage *k*-mer connecting tips were kept by using the –keep-mercy (-y) argument, and the compacted de Bruijn graph was colored using the –colors (-c) argument.

Our initial intent was to utilize the de Bruijn graph structure in the downstream analysis, but the compacted de Bruijn graph for *k* = 9 is almost complete: It contains 131,070 nodes, i.e. *k*-mers and 1,048,544 in-edges, resulting in >7.99 edges per node (similarly >7.99 for out-edges). Note that the maximum in-degree and out-degree in the compacted graph is 8, not only 4, since each node can be traversed in forward and reverse complement direction (Holley and Melsted 2020). We, therefore, focused purely on the *k*-mers.

The resulting *k*-mers were then filtered according to 2 aspects. First, the expression of those Alu elements possessing the *k*-mer was compared with those not possessing it. Second, for any given *k*-mer, its suffixes and prefixes were generated. Each *k*-mer possesses 4 potential pre- and suffixes, as each sequence can be preceded or succeeded by one of the 4 bases.

When testing a *k*-mer, say XJY, with X,Y∈{A,C,T,G} and J a fixed 2-mer, for having an effect on Alu transcription, we have to guard against false positive due to the large number of *k*-mer tests. This is done by Bonferroni multiple testing correction for a family-wise error rate of at most 5%. We further need to filter out irrelevant findings resulting from the large number of observations that can render even small differences highly significant. To ensure that XJY is relevant for Alu transcription, we compare the group of Alu elements containing XJY to the group of Alu elements that contain the prefix XJ or the suffix JY, but not the full *k*-mer XJY. We compare the 2 groups with respect to their binarized expression (expressed with at least 1 read count/not expressed) using a Fisher test, and we require an OR of at least 2 or at most 0.5 for being considered a relevant difference.

## Results

### Alu expression increases with family age

Many Alu families that have emerged in the human genome over the last 65 million years vary not only in their sequence characteristics (Jurka and Smith 1988, but also in their activity (Bennett *et al.* 2008; Oler *et al.* 2012); active Alu elements being those that are not only transcribed but are also mobile have the capability to reinsert themselves into the genome through retrotransposition.

**Fig. 2.**
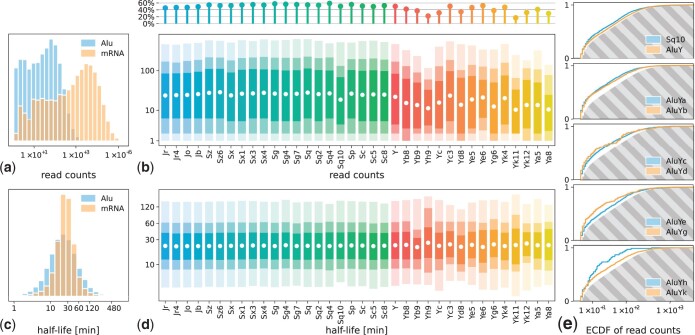
Alu expression and half-life—all subfigures based on data published by Schwalb *et al.* (2016), using the UCSC Genome Browser Annotation. a) Histogram of the read counts for both Alu elements and mRNA; Alu elements show less transcriptional activity overall (histogram *y*-axes: density). b) Read count distribution for all Alu families as shown in Fig. 1b. The colored bars represent the extent of the 50%, 75%, and 95% median percentile interval with decreasing opacity. The white dot indicates the arithmetic mean. Zero counts are disregarded in this subfigure. The upper lollipop plot shows the proportion of Alu elements per family with more than 0 read counts. c) Histogram of the half-life of both Alu elements and mRNA; Alu elements show stability comparable to regular transcripts. d) Half-life distribution for all Alu families (cf. Fig. 1b), binned by family. e) ECDF of the read counts of younger Alu families (AluY and subsequent, as well as AluSq10) in contrast to all older families, represented by the gray-shaded area. Younger Alu families show consistently lower read counts in comparison to the older ones.

As was found by Bennett *et al.* (2008) in vitro, the activity of an Alu family appears to scale inversely with its age; the older an Alu element, the less likely it is to be still capable of successful reinsertion. We examined the transcriptional activity of Alu families, using the RNA-seq data from Schwalb *et al.* (2016) (see *Methods*), as well as the stability of Alu transcripts, which is expected to be low in comparison to regular mRNA (An *et al.* 2004 and Fig. 2, a–e). We used maximum likelihood estimation (MLE) based on metabolic RNA labeling to calculate the half-life $t_{1/2}$ of Alu RNAs (see *Methods*), which relates to the degradation rate *δ* by $t_{1/2}=\ln\left(2\right)/\delta$, as further detailed in Supplementary material S5.

The global expression distribution of Alu transcripts (mean $\bar{x}\approx 85$, median $\tilde{x}=27$) is about 2 orders of magnitude lower than that of mRNAs ($\bar{x}\approx 4088,\;\tilde{x}=740$), as is to be expected (Fig. 2a, Paulson and Schmid 1986). We found that members of the younger Alu families, mostly AluY, but also AluSq10, exhibit overall less transcriptional activity than older Alus (Fig. 2b). This effect is most pronounced with the subfamilies of AluY. Since the differences between the heavy-tailed read count distributions are not easy to visualize, we compare the empirical cumulative distribution functions (ECDF) of the young Alu families with the ECDF of the old ones (Fig. 2e). All young ones are significantly different from the old ones [*P* < 0.05 in all comparisons, Kolmogorov–Smirnov (KS) test or Wilcoxon test, both with Bonferroni multiple testing correction]. The split can be further verified by performing average linkage hierarchical clustering with the KS test statistic as a distance measure between Alu families. This separates the AluY subfamilies clearly from the older families, except for the aforementioned AluSq10 and AluYc3 (AluYc2) (Supplementary material S7a).

The use of TT-seq (Schwalb *et al.* 2016) and 4sU-seq (Miller *et al.* 2011) allows for the identification of newly created transcripts, and thereby, the estimation of RNA synthesis and degradation rates, and finally half-lives (see *Methods*). However, due to the overall paucity of Alu read counts, our MLE results are relative and do not represent explicit values (see *Discussion*). Our half-life estimates for Alu elements show a dispersion very similar to that of mRNAs (Fig. 2c). This result is surprising, as the in silico study by An *et al.* (2004) suggests that Alu transcripts should be notably less stable than mRNAs. In addition, the half-life distribution of Alu RNAs remains similar between families, even in the youngest subfamilies of AluY that have only a few members and are generally lowly expressed (Fig. 2d).

### Alu expression is independent of gene transcription

Alu elements are often presumed to be Pol-III transcripts, as they contain a bipartite Pol-III promoter (Orioli *et al.* 2012). Although there is experimental evidence for Alu elements being transcribed by Pol-III (Jagadeeswaran *et al.* 1981; Panning and Smiley 1993; Zhang *et al.* 2019), it has not been ruled out yet that (some) Alu elements might be transcribed by RNA Pol-II. We have therefore examined the possibility that Alu transcripts may also arise as side products of RNA Pol-II gene transcription, as hinted at by the results of Conti *et al.* (2015) and suggested by Zhang *et al.* (2019).

If Alu elements are also transcribed by Pol-II as side products of gene transcription, we would expect intragenic Alu elements to exhibit a higher transcript abundance than intergenic ones. Furthermore, the transcriptional activity of genes should be correlated with that of proximal Alu elements. The expression of those should be biased toward Alu elements lying in sense direction with regard to their associated gene.

To investigate this, we defined intragenic Alu elements as those that either directly overlap a gene or lie within 500-bp up- or downstream of a gene according to the GRCh37/hg19 canonical gene annotation of the UCSC Table Browser (Karolchik *et al.* 2004). All other Alu elements are called intergenic. Over 96% of intragenic Alu elements overlap intronic regions according to the latest GENCODE annotation (Frankish *et al.* 2021). Effects of proximity to Pol-I and Pol-III transcripts have not been detected and cannot contribute a notable effect, since only a comparably tiny number of Alu elements would be affected. Of the expressed Alu elements, 37 loci lie in 500-bp proximity to rRNAs (excluding 5S rRNA), and 42 in proximity to tRNAs, using the respective UCSC Table Browser annotation.

**Fig. 3.**
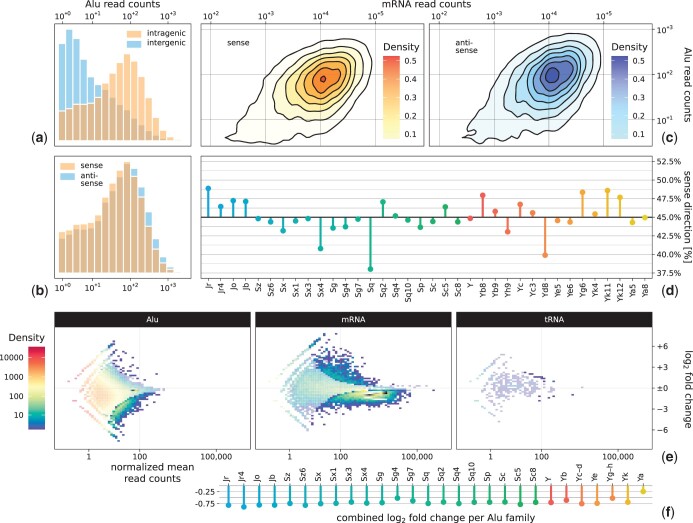
Alu correlation and differential expression. a) Histogram of the read counts for transcribed intragenic (398,660) and intergenic (200,928) Alu elements. Intragenic Alu elements either directly overlap a gene or lie within 500-bp up- or downstream of a gene. Other Alu elements are intergenic. Intragenic Alu elements show a significantly higher expression than intergenic ones, which is likely explained by genome accessibility. b) Histogram of the read counts for transcribed intragenic Alu elements that lie in sense or antisense direction with respect to their associated gene, showing only minor differences. c) 2D kernel density estimation showing the correlation between the annotated Alu and mRNA read counts for intragenic sense (orange) and antisense (blue) Alu elements. The 2 groups show no difference in correlation strength. d) Number of sense and antisense Alu elements per family. No family exceeds a ratio of 38% or 50%. e) 2D density heatmap showing the DESeq2 differential expression of Alu elements, mRNAs, and tRNAs as control under α-amanitin Pol-II inhibition. Semitransparent areas do not pass the significance threshold. Loci with a normalized mean expression below 0.1 are not shown, with affects 51% of all annotated Alu loci and practically no mRNAs or tRNAs. Both Alu elements and mRNAs show stronger significant downregulation than upregulation. f) Differential expression of Alu elements under α-amanitin Pol-II inhibition, using family-wise aggregated read counts. All Alu families show downregulation.

We compared the read counts for intragenic and intergenic Alu transcripts, which show a significantly higher expression for intragenic Alus compared to intergenic ones (Fig. 3a, $\bar{x}\approx 110,\;\tilde{x}=49$ intragenic, $\bar{x}\approx 35,\;\tilde{x}=6$ intergenic, $P<10^{-5}$, Mann–Whitney test). We also detected a less pronounced but significant difference in the expression of intragenic Alu elements lying in sense respectively in the antisense direction with regard to their associated gene (Fig. 3b, $\bar{x}\approx 97,\;\tilde{x}=44$ sense, $\bar{x}\approx 121,\;\tilde{x}=54$ antisense, $P<10^{-5}$, Mann–Whitney test).

We also correlated the transcript abundance of intragenic Alu elements and their associated genes for both Alu elements in the sense and antisense direction to their associated gene. The 2 groups show no difference in correlation strength (Spearman correlation $r\approx 0.71,\;P<10^{-5}$ rand. test, Fig. 3c).

Lastly, we examined the number of sense and antisense Alu elements per family. Overall, 46% of all intragenic Alu elements lie in the sense direction with regard to their associated gene. No individual family exceeds a ratio of either 38% or 50% (Fig. 3d).

Furthermore, we combined Pol-II and Pol-III occupancy data drawn from the ENCODE portal (ENCSR000EHL, ENCSR000EHQ, Dunham *et al.* 2012; Davis *et al.* 2018) with our RNA-seq data and RNA-seq data from ENCODE (ENCSR000COM). While we found a modest correlation between our own sequencing’s mRNA transcript abundance and the data obtained from ENCODE (Spearman correlation r≈0.65), our analysis did not detect global correlation between Pol-II or Pol-III occupancy and individual Alu expression according to our sequencing data (Spearman correlation r≤0.05 for both, Supplementary material S7b).

### RNA Pol-II inhibition decreases Alu expression

To further examine the origin of Alu transcripts, we performed a Pol-II inhibition experiment by incubating K562 cells with α-amanitin at a concentration suited to efficiently inhibit the Pol-II activity while leaving the activity of Pol-III and mitochondrial RNA polymerases unaffected (see *Methods*). We quantified the degree of inhibition using mitochondrial RNAs as a negative control group, which are unaffected by α-amanitin (see *Methods*). Similar results were obtained when spike-in RNAs were taken as reference (Supplementary material S7c). The analysis of tRNAs as an independent negative control group showed barely any differential expression, validating that our normalization procedure did not introduce a bias to putatively unaffected transcripts. As a natural positive control group, we chose mRNAs that are bona fide Pol-II transcripts. Figure 3e shows the changes after α-amanitin inhibition for Alu elements, mRNAs, and tRNAs.

Due to the low coverage of individual Alu transcripts, a direct comparison based on individual expression folds would be misleading. We, therefore, aggregated all read counts mapping to 1 Alu family (or several families in the case of very small families, Fig. 3f) and calculated the expression fold of each Alu family. All Alu families show downregulation with an average log_2_ fold change (l2fc) of −0.70 (standard deviation σ≈0.12, min=−0.88 for AluYr4, max=−0.24 for AluYa). In comparison, individual mRNAs with at least 10 reads exhibit a downregulation of −0.35 (σ≈0.99). Also, Alu elements being intragenic or intergenic show no influence on their response to α-amanitin (>90% downregulated among significantly differentially expressed transcripts in both cases); therefore, these 2 groups are treated as 1 in the following.

**Fig. 4.**
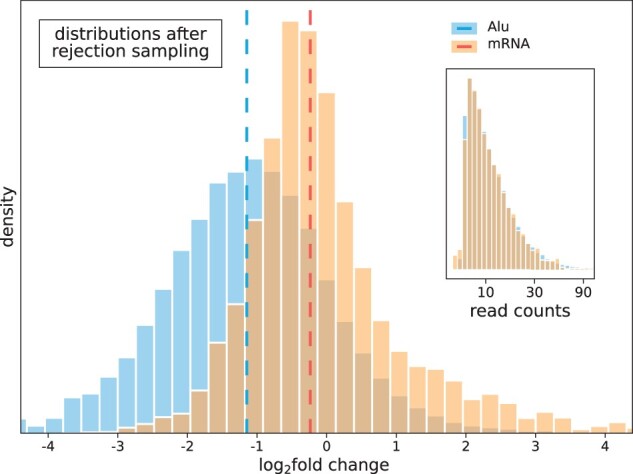
Rejection sampling—distribution of DESeq2 differential expression of Alu elements and mRNAs after rejection sampling according to transcript length corrected Alu normalized mean read counts (shown in inset). Vertical lines denote median Alu l2fc of −1.15 (blue), and median mRNA l2fc of −0.24 (red). Alu elements exhibit downregulation comparable to canonical genes under α-amanitin Pol-II inhibition.

As the overall mean expression of Alu elements differs strongly from that of mRNAs (cf. Fig. 2a), we used rejection sampling to obtain comparable l2fc distributions (Fig. 4, Flury 1990). Alu and mRNA differential expression appears very similar, with the distribution of Alu l2fc falling even below that of mRNA. Examining individual l2fc values, we focused on those Alu elements or mRNAs with significant differential expression (P≤0.05, Wald test with Benjamini–Hochberg multiple testing correction). Of all Alu elements, 16,909 (89%) of 19,027 transcripts show no significant differential expression while 2,118 (11%) do. A total of 9,818 (53%) of 18,590 mRNAs show no significant differential expression while 8,772 (47%) do. tRNAs are largely unaffected by α-amanitin, with only 17 of the total 604 (3%) tRNA transcripts exhibiting significant change, 12 being up- and 5 being downregulated. This is to be expected, as tRNAs are transcribed by Pol-III (White 1997).

Alu elements, while expectedly represented with considerably less read counts than mRNA transcripts, still show downregulation under α-amanitin treatment, with an average l2fc downregulation of −3.23 (σ≈1.24) for significant transcripts. Accordingly, mRNAs exhibit an average significant downregulation of −0.99 (σ≈0.43).

However, both Alu elements and mRNAs show a small fraction of significantly upregulated transcripts under α-amanitin inhibition of Pol-II. In comparison to the significantly downregulated fraction though, this fraction is small with 1,002 (5%) significantly upregulated but 7,770 (42%) significantly downregulated transcripts for mRNAs and 47 (0.2%) significantly upregulated but 2,071 (11%) significantly downregulated transcripts for Alu elements.

It is remarkable that some Alu elements show significant downregulation under α-amanitin treatment at all, which suggests that the affected Alu elements could be partly Pol-II transcripts.

### Sequence features associated with Alu expression show signs of evolutionary selection

Evolutionary changes in the Alu sequence could reveal informative clues on sequence features that influence the mechanism of Alu transcription and retrotransposition. Obvious, prominent features of potential impact are the Pol-III promoter split into A and B box and the SRP-binding motif used for attachment to the ribosome exit tunnel (see *Introduction*).

**Fig. 5.**
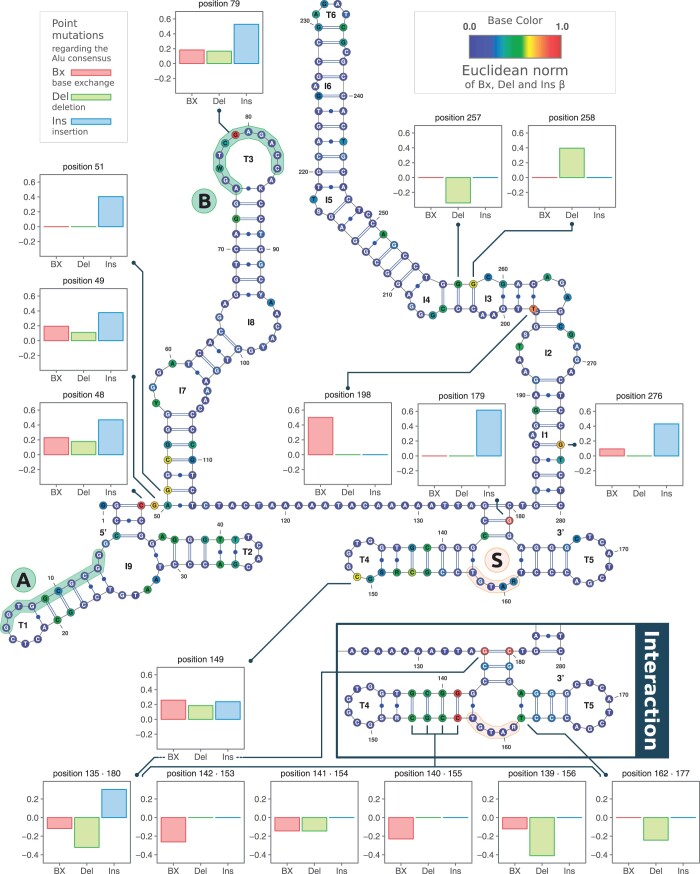
GLM and Alu secondary structure—secondary structure of the Alu element consensus sequence according to Sinnett *et al.* (1991), showing the left and right arm architecture with the variable region in between. Noteworthy sequence features include the bipartite Pol-III promoter split into A and B boxes (denoted by Ⓐ and Ⓑ), and the SRP-binding motif used for attachment to the ribosome exit tunnel (denoted by Ⓢ). Each base is colored according to the rescaled Euclidean norm of the 3 GLM effect sizes (β parameters). Several noteworthy positions are annotated with bar graphs detailing the individual values. In the lower right corner, prominent results from the analysis of interactions between paired bases are shown.

The abundance of Alu sequences in the genome offers the unique opportunity to use an analysis strategy resembling a genome-wide association study. We used a GLM to link Alu sequence features to their transcript abundance (see *Methods*). Due to the encoding of the sequence changes with respect to the Alu consensus sequence, this results in individual effect sizes (β parameters) for the 3 types of examined point mutations, base exchanges, deletions, and insertions, for each position in the Alu consensus sequence. Figure 5 shows the Alu secondary structure according to Sinnett *et al.* (1991) with the Euclidean norm of the effect sizes represented by base color (dot-bracket notation of the secondary structure in Supplementary material S8). However, the large number of available Alu sequences enabling this analysis also precludes any attempts to test for statistical significance.

Several noteworthy positions show a high correspondence to Alu transcription (Fig. 5): Position 48 is sensitive to all 3 types of point mutations, but primarily to insertions. It lies in the left arm at the juncture between inner loop 7 (I7) and inner loop 9 (I9), which contains the A box of the Pol-III promoter sequence. Position 79, also in the left arm in terminal loop 3 (T3), lies directly within the B box of the Pol-III promoter sequence. Like the previous position, it is sensitive to all 3 types of mutation, but primarily to insertions, which would bring the Alu consensus sequence closer to the consensus sequence of the tRNA promoter: GWTCRANNC (Paolella *et al.* 1983). Position 179 lies in the right arm near terminal loops 4 and 5 (T4, T5) and reacts only to insertions, and position 198, also in the right arm between inner loops 2 and 3 (I2, I3), reacts only to base exchanges.

Due to the large number of samples (i.e. Alu elements), we could also investigate interactions between paired bases in the secondary structure (see *Methods*), which resulted in 2 positions of interest, all in the vicinity of terminal loops 4 and 5 (T4, T5) of the right arm, which is also the region containing the SRP 9/14-binding motif: UGU(NR) (Weichenrieder *et al.* 2000). The pair of positions 135 and 180 reacts inversely to insertions in comparison to base exchanges and deletions. It forms the junction from which terminal loops 4 and 5 (T4, T5) branch off. The pair of positions 139 and 156 lies directly adjacent to the SRP-binding motif and reacts primarily to deletions, as well as to base exchanges.

As the GLM focused on individual bases or base pairs in the Alu consensus sequence, we also developed our own *k*-mer-based method to search for sequence motifs that influence Alu expression, better suited to the data at hand than regular motif search. First, to circumvent the problems that arise from the use of a reference sequence, we constructed a de Bruijn graph from the *k*-mers of all Alu sequences (k=9, for the tuning of *k* and its justification, see *Methods*). Next, we filtered for *k*-mers with statistical significance and biological relevance. Each *k*-mer splits the group of all Alu sequences into those containing the *k*-mer and those that do not. At the same time, each Alu element can be either expressed (at least 1 read count) or not. This gives rise to a 2 × 2 contingency table, to which we apply a Fisher test and calculate the odds ratio (OR, see *Methods*). *k*-mers with both an OR of less than 0.5 or greater than 2 and a Bonferroni-corrected Fisher test *P*-value less than 0.05 were included in the downstream analysis, leaving us with 38 *k*-mers of interest. Finally, we performed another Fisher test comparing expressed and nonexpressed Alu elements in the group containing a specific *k*-mer of interest vs the group of Alu elements containing the *k*-1 Prefix and suffix of that *k*-mer. This resulted in merely 12 remaining *k*-mers of interest after applying the same stringency criteria (Table 1). We also ensured that the reported *k*-mers originate from the Alu sequence itself and not from the 100-bp flanking region (see *Methods* and Supplementary material S11).

**Table 1.** De Bruijn graph significant *k*-mers.

	*k*-mer	OR	JASPAR match	Matrix ID
1	AACGCGCCA	2.65	—	
2	ATCGCCCGC	2.72	**NFIX**	MA0671.1
			NR2C2 (var.2)	MA1536.1
3	CGGACTGCT	2.07	MEIS1	MA0498.2
			TEAD3	MA0808.1
4	CTCAACGCC	2.40	SOX18	MA1563.1
			BARHL1	MA0877.2
			ZNF354C	MA0130.1
			NR2C2 (var.2)	MA1536.1
			GSX2	MA0893.2
5	GAAACCGTC	2.12	—	
6	GACACGCGC	2.27	**ARNT::HIF1A**	MA0259.1
			**TFE3**	MA0831.2
			USF1	MA0093.1
7	GATCGCCCG	2.56	GATA2	MA0036.1
8	GGCGGACTG	2.35	MEIS1	MA0498.2
9	GGGCGGACT	2.64	—	
10	TAGGCGCGC	2.08	—	
11	TCAACGCCT	2.22	TBX4	MA0806.1
			TBX5	MA0807.1
			NR2C2 (var.2)	MA1536.1
			GSX2	MA0893.2
			MGA	MA0801.1
12	TGACACGCG	2.92	FOS::JUN	MA0099.2
			**TFE2**	MA0831.2
			TBX4	MA0806.1
			MGA	MA0801.1
			TBX5	MA0807.1
			USF1	MA0093.1

Shown are the *k*-mers (k=9) from the de Bruijn graph created of all Alu sequences (incl. 100-bp flanking regions) that passed all significance and relevance thresholds: P≤0.05 (Fisher test with Bonferroni multiple testing correction) and OR ≤ 0.5 or OR ≥ 2.0 for both test schemes. Listed are the *k*-mer sequence, associated OR (*k*-mer occurrence vs read counts), JASPAR match, and associated Matrix ID (see Supplementary material S10 for expanded statistics). The binding profile matches described in the *Results* are marked as bold.

A comparison of those *k*-mers with transcription factor-binding profiles from the JASPAR database reveals that 8 of the 12 *k*-mers show similarities to known motifs (Fornes *et al.* 2020). The most intriguing findings to us were the *k*-mers resembling the binding profiles of ARNT::HIF1A (MA0259.1, which is a master transcriptional regulator of hypoxia response), NFIX (MA0671.1, which is involved in the replication of adenovirus 2), and TFE3 (MA0831.2, which plays a role in the general immune response). Also, all listed transcription factors are Pol-II specific (see Supplementary material S10 for references and detailed statistics).

## Discussion

We presented a multifaceted analysis of the Alu elements found in the human genome in K562 cells, focusing on their RNA metabolism and sequence features that influence it.

The read count distributions of Alu elements in comparison to mRNA presented in Fig. 2, a, b, and e correspond well to previous in vitro findings, corroborating that Alu expression is generally low in comparison to gene transcription and that the younger Alu families (AluY and subfamilies) are expressed at even lower rates than the older Alu families (AluJ and AluS) (Paulson and Schmid 1986; Bennett *et al.* 2008).

In contrast, the results of our RNA half-life estimation of Alu transcripts through the use of TT-seq (Schwalb *et al.* 2016) and 4sU-seq (Miller *et al.* 2011) are surprising. Previous studies suggest that Alu RNAs should be less stable than regular gene transcripts, as they contain adenine- and uracil-rich element motifs, a sequence feature linked to decreased transcript stability with an as yet unknown mechanism (An *et al.* 2004). In contrast, our MLE predicts that Alu transcripts and mRNAs should be of similar stability (Fig. 2, c and d). However, our MLE can only serve as an assessment to compare the relative half-life distributions; its predictions do not represent explicit half-life values. First, the MLE has to contend with the overall paucity of Alu read counts, which limits its explanatory power, an issue that could only be remedied by vastly increasing sequencing depths in future experiments. Second, to not exacerbate the already demanding estimation, our model contains some simplifications, such as the assumption of steady-state conditions, using a Poisson distribution to model read counts instead of a zero-inflated negative binomial distribution, and neglecting nonconstant labeling efficiencies for short labeling periods (see Supplementary material S5). As such, our half-life estimates point toward a need for further research regarding the stability of Alu transcripts, as the subject matter is less straightforward as it appears prima facie. Also, our data originate from K562 cells and are thus subject to the peculiarities of that cell line (Li *et al.* 2000). Nonetheless, taking into account the nonautonomous life cycle of Alu elements and their reliance on LINE-1 repeat translation (Boeke 1997; Dewannieux *et al.* 2003) in conjunction with their low expression, transcript persistence in the cell could potentially be instrumental for successful retrotransposition.

Regarding our noninterventional analysis concerning the origin of Alu transcripts, we observed a higher expression of intragenic Alu elements compared to intergenic ones (Fig. 3a). This on its own cannot be taken as solid evidence either for or against a connection between Alu and gene transcription. Genome accessibility is a confounding factor, as intragenic regions are in general more accessible than intergenic ones (Guo *et al.* 2017). It should also be noted that a screening of Alu elements proximal to Pol-I and Pol-III transcripts, while intriguing, falls outside the scope of our study, as our approach exploits the large group size of intraend intergenic Alu elements to compensate for the low read counts. The difference in expression between intragenic Alu elements that lie in sense or antisense direction with respect to their associated gene is so minor that we deem it irrelevant (Fig. 3b). Therefore, we analyzed the correlation between Alu and gene transcription (Fig. 3c). A certain level of correlation is to be expected regardless of Alu transcript origin due to genome accessibility, as mentioned above. However, the lack of a difference in correlation strength between sense and antisense points toward Alu transcripts not originating primarily as side products of Pol-II gene transcription. If that were the case, a stronger correlation between Alu elements in sense direction with respect to their associated gene and the expression of that gene should exist. This is further corroborated by the number of sense and antisense Alu elements present in each family (Fig. 3d). If Alu transcripts were primarily side products of Pol-II gene transcription, Alu insertion should show a bias toward aligning new Alu elements in sense direction with respect to their associated gene. Taken together, we interpret these findings as weakening the hypothesis that Alu transcripts are mainly created alongside Pol-II gene transcription. This does not rule out the possibility for a fraction of Alu elements being transcribed in this fashion, but it does not appear to be a major contributor to Alu expression in the cell.

Our analysis of Pol-II and Pol-III occupancy data did not detect any correlation between Alu expression and polymerase occupancy (Supplementary material S7b), which was a likely outcome given the nonconformity of the 2 measurements (Ehrensberger *et al.* 2013; Gressel *et al.* 2017). The issue of such a correlation was also addressed recently by Zhang *et al.* (2019), who utilized an extensive collection of data produced as part of the ENCODE project in conjunction with RAMPAGE (Batut and Gingeras 2013). While their study covered only 1.5% of all annotated Alu elements in the human genome due to strict read-mapping and analysis constraints, they identified a subset of Alu elements as Pol-III transcribed, also in accordance with chromatin immunoprecipitation-seq data. This suggests that the detection of correlation between Alu expression and polymerase occupancy, while possible, necessitates high-resolution data that make a global assessment demanding and beyond the explanatory power to be found in our data.

The differential expression analysis under α-amanitin inhibition of Pol-II suggests that a substantial part of Alu transcription is Pol-II dependent (Fig. 3, e and f). To the author’s knowledge, this is the first direct experimental evidence for the origin of Alu transcripts. If Alu transcripts would originate mainly from the Pol-III activity, no downregulation should be present. While this may appear to conflict with our previous findings that Alu expression is unlikely to be primarily a side product of Pol-II gene transcription, this leaves the possibility open that Alu transcripts arise from Pol-II activity independent from gene transcription. However, it seems more likely that Alu RNAs arise from different modes of transcription simultaneously, as also suggested by Conti *et al.* (2015) and Zhang *et al.* (2019). This is of particular interest regarding differential expression analyses of the past that utilized Pol-II inhibition, as it is common practice to rely on SINEs as a non-Pol-II-dependent negative control group in such experiments. Alus are the most common SINE in the human genome (Cordaux and Batzer 2009), which may call the results of these studies into question. Regarding our own results, the small but not negligible fraction of seemingly upregulated Alu and mRNA transcripts may be explained by inhibition of nuclear export (Bahar Halpern *et al.* 2015; Zander *et al.* 2016). To achieve noticeable differential expression of Alu elements under α-amanitin inhibition of Pol-II, an incubation time of 8 h was required (see *Methods*). This exposes the cells to extreme levels of stress, as their protein metabolism is breaking down. This, in turn, may lead to the retention and accumulation of RNAs that are not related to shock response in the nucleus. As our DTA does not differentiate between nuclear and cytoplasmic RNA fractions, this could appear as apparent upregulation. Alternatively, protein translation inhibition, especially of proteins related to mRNA degradation (such as deadenylases or Xrn1), could be involved (Orban and Izaurralde 2005; Dobin *et al.* 2016). Changes in protein level, even after extended exposure, seem to be an uncommon effect of α-amanitin treatment, according to Fiume and Stirpe (1966) and Stirpe and Fiume (1967). A reduction in protein abundance or concentration would approximately lead to a proportional decrease in degradation rates. Hence, it would merely lead to a proportional shift in total expression levels. The reduction in the protein concentration might therefore lead to compensatory effects, which would make a quantitative, but not a qualitative change. Regarding Fig. 3e and f it should also be noted that an average l2fc of −0.35 (σ≈0.99) for mRNAs may appear small when considered individually, especially in the 2D density heatmap, where strongly expressed genes do appear to be affected more, as random measurement noise is visually overrepresented and optically dominates the plot. However, calculating the overall differential expression shows a global reduction in mRNA levels by a factor of 3 (l2fc of −1.64), comparable to, for example, the effect of heat shock response (Mahat *et al.* 2016).

One influential position resulting from our GLM analysis of all Alu sequences is of particular note (see Fig. 5). Position 79 lies within the B box of the Pol-III promoter sequence and is most sensitive to insertions, which bring it closer to the consensus sequence of the tRNA promoter: GWTCRANNC (Paolella *et al.* 1983). This suggests that the promoter plays a role in Alu transcription, further substantiating the assumption that Alu RNAs arise from various methods of transcription.

It is remarkable that our new method employing de Bruijn graph *k*-mers does detect statistically significant and biologically relevant sequence motifs when previous attempts using regular *de novo* motif search did not (Zhang *et al.* 2019). We attribute this to some extent to the tuning of the parameter *k* (see *Methods*). We found that with a *k* either smaller or larger than 9, the high sequence similarity of Alu elements quickly leads to problems. With a *k* smaller than 9, many *k*-mers are possessed by all or almost all Alu elements, while with a *k* larger than 9, a high number of *k*-mers are unique to a few or even a single Alu sequence. In both unfavorable cases, a majority of *k*-mers are ruled out simply by the selection of *k*. This might appear as a limitation of our method, but in fact, it is an intrinsic limitation shared by all continuous-motif-based methods. Our method merely makes this problem noticeable and offers a solution through parameter tuning. We also see the potential for our approach to be applied to other data sets in the future.

Taken together, we present a compendium of results regarding the RNA metabolism of Alu elements. Our analyses affirm that older Alu families are more strongly expressed than younger Alu families, but the stability of Alu transcripts appears to be close to that of mRNA. Furthermore, we find evidence for Alu transcripts originating both from Pol-II and Pol-III activities, but no evidence for Alu transcripts being side products of Pol-II gene transcription. Finally, we have identified a list of sequence features that influence Alu transcription and are thus targets for further investigations and developed a novel method to test the statistical significance and biological relevance of de Bruijn graph *k*-mers.

## Data availability

The sequencing data used in this publication have been deposited in NCBI’s Gene Expression Omnibus (Edgar *et al.* 2002) and are accessible through GEO Series accession number GSE75792 (https://www.ncbi.nlm.nih.gov/geo/query/acc.cgi?acc=GSE75792) for the data described in Schwalb *et al.* (2016), and through GEO Series accession number GSE185485 (https://www.ncbi.nlm.nih.gov/geo/query/acc.cgi?acc=GSE185485) for the data relating to the inhibition of RNA Pol-II by α-amanitin. GSE75792 was used for all non-interventional analyses, while GSE185485 was used for the differential expression analysis.

Supplemental material is available at *G3* online.

## Supplementary Material

jkac054_Supplement_S1

jkac054_Supplement_S2

jkac054_Supplement_S3

jkac054_Supplement_S4

jkac054_Supplement_S5

jkac054_Supplement_S6

jkac054_Supplement_S7

jkac054_Supplement_S8

jkac054_Supplement_S9

jkac054_Supplement_S10

jkac054_Supplement_S11
