# Supplementary material for: RNA transcription and degradation of Alu retrotransposons depends on sequence features and evolutionary history
Source: G3 (Bethesda). 2022 Mar 7;12(5):jkac054. doi: 10.1093/g3journal/jkac054 (PMC9073682; doi:10.1093/g3journal/jkac054)
Supplement: jkac054_Supplement_S2 [file jkac054_supplement_s2.pdf]

Table S2. Real-time reverse transcription polymerase chain reaction (RT-qPCR) primers used in this study. Primer sequences were designed using Primer3 software, following the parameters described in the **Methods** section. PCR efficiency values of primers (E) were calculated for each gene from the given slope after running standard curves.

Ref. #	Direction	Primer sequences	Length (nt)	Primer efficiency (E)	Target	Polymerase (Pol)
**SG-116**	forward	ACAATTCCAAATAGCGACCACATCA	25	0.97	Synthetic, ERCC-00043 („Spike-in 2“)	-
**SG-117**	reverse	TACCTCAACCCTTCCAGTGTCTAAG	25			
**SG-118**	forward	CATAAGCGGAGAAAGAGGGAATGAC	25	1.06	Synthetic, ERCC-00145 („Spike-in 5“)	-
**SG-119**	reverse	GCTAAATAGAGAGCATCCACACCTC	25			
**MM-fw**	forward	AGACTGGCATTCCCGTGATA	20	1.00	Synthetic, ERCC-00170 („Spike-in 12“)	-
**MM-rev**	reverse	GCTAAAACCCCTGCCTGCAA	20			
**SG-104**	forward	TGTCTGTCCTACTACCATGTCTGAA	25	1.00	Human, CWC22	Pol II
**SG-105**	reverse	TCCATATAAAGTGCCAAGGGTTCAC	25			
**SG-84**	forward	TCTCTGTTTGGAAGCCATTTGACTC	25	1.07	Human, PAIP1	Pol II
**SG-85**	reverse	AAAGCCTGCATTACTTCTCTAGCAC	25			
**SG-98**	forward	GGATTCTCCGTATTTGCGTCAGC	23	1.03	Human EGR1	Pol II
**SG-99**	reverse	GCTACCATTGACTCCCGAGGTA	22			
**SG-126**	forward	GTAACCCGTTGAACCCCATT	20	1.10	Human, 18S rRNA	Pol I
**SG-127**	reverse	CCATCCAATCGGTAGTAGCG	20			
**SG-130**	forward	CTCGCTTCGGCAGCACA	17	1.15	Human, U6 snRNA	Pol III
**SG-131**	reverse	AACGCTTCACGAATTTGCGT	20			
