# Supplementary material for: RNA transcription and degradation of Alu retrotransposons depends on sequence features and evolutionary history
Source: G3 (Bethesda). 2022 Mar 7;12(5):jkac054. doi: 10.1093/g3journal/jkac054 (PMC9073682; doi:10.1093/g3journal/jkac054)
Supplement: jkac054_Supplement_S3 [file jkac054_supplement_s3.pdf]

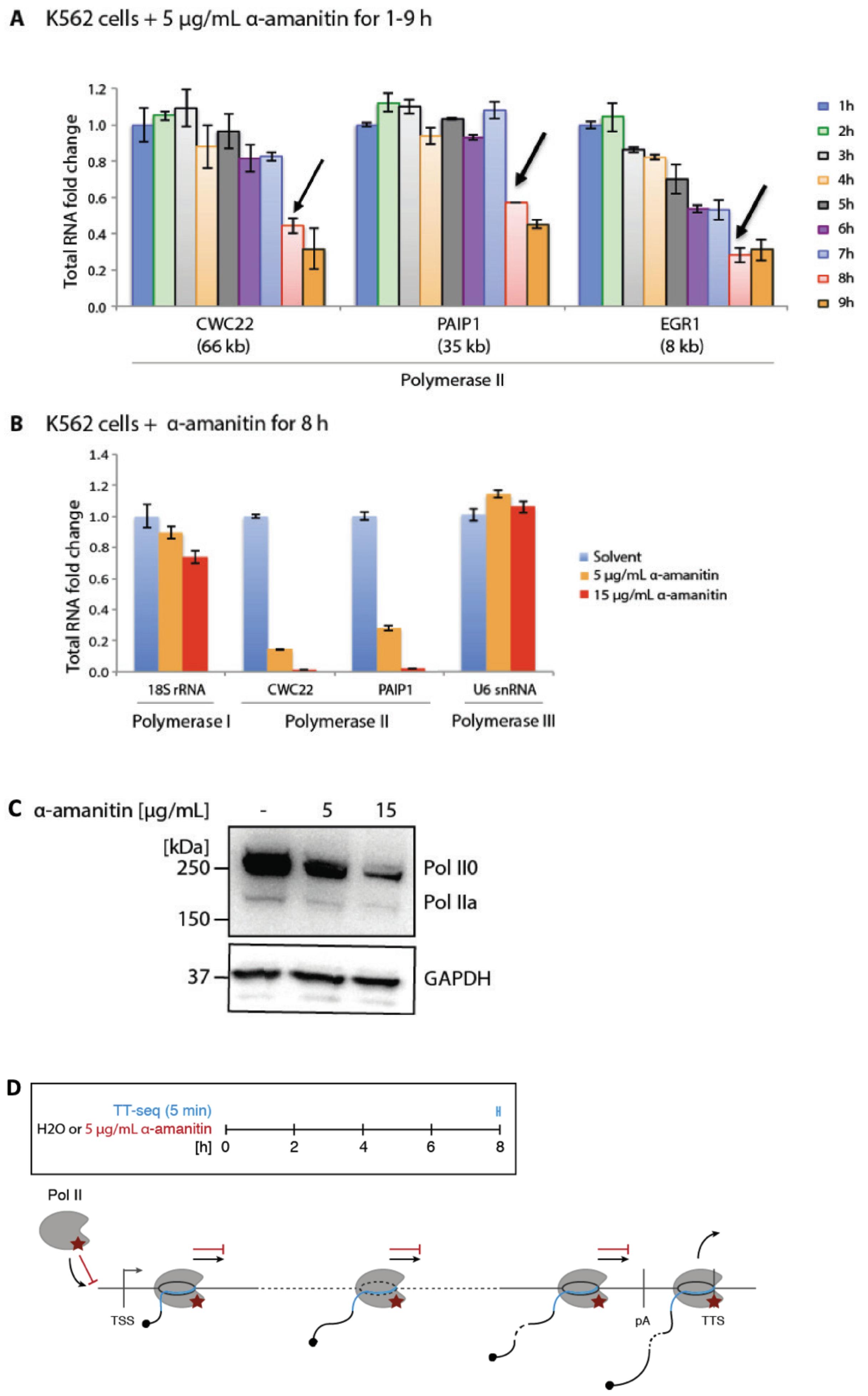

Figure S3 Supplemental figures for quality control. Treatment conditions were optimized for selective Pol-II inhibition. **a-b**) Relative expression levels of genes were analyzed using RNA spike-ins for qPCR normalization in K562 cells treated with α-amanitin (5 or 15 μg/mL) versus solvent control. The 2^(-ΔΔ Ct) method was applied to calculate the normalized target gene expression fold change, with the amplification efficiency (E) for each target gene, slope of standard curve (S) and mean threshold cycle (Ct) (Livakand Schmittgen 2001). Bars represent the means and standard errors of two technical replicates. **a**) The time point (8 h) at which about 50% decrease was observed for all Pol-II genes (CWC22, PAIP1, EGR1) is indicated by arrows. **b**) Relative expression levels of Pol-I (18S rRNA), Pol-II (CWC22, PAIP1) and Pol-III (U6 snRNA) genes were analyzed as described above. K562 cells were treated for 8 h with 5 or 15 μg/mL α-amanitin or solvent. At low α-amanitin concentrations (5 μg/mL), Pol-III and Pol-I expression levels remained close to levels in control conditions while Pol-II transcript levels decreased substantially. **c**) Degradation of the largest subunit of Pol-II, POLR2A (hRPB1) is α-amanitin dose dependent. At low α-amanitin concentrations (5 μg/mL), POLR2A levels remained close to levels in control conditions which agrees with published data (see, e.g., Fig. 1A in Nguyen et al.1996). Western blot analysis after treatment with 5 or 15 μg/mL α-amanitin for 8 h. POLR2A (bands correspond to the phosphorylated, IIo, and unphosphorylated, IIa forms of POLR2A) was visualized by N-terminal antibody F-12 (top panel). GAPDH was used as loading control (bottom panel). **d**) Graphical representation of the experimental design. TT-seq was carried out with K562 cells after treatment with solvent (water) or α-amanitin (5 μg/mL).
