# Supplementary material for: RNA transcription and degradation of Alu retrotransposons depends on sequence features and evolutionary history
Source: G3 (Bethesda). 2022 Mar 7;12(5):jkac054. doi: 10.1093/g3journal/jkac054 (PMC9073682; doi:10.1093/g3journal/jkac054)
Supplement: jkac054_Supplement_S4 [file jkac054_supplement_s4.pdf]

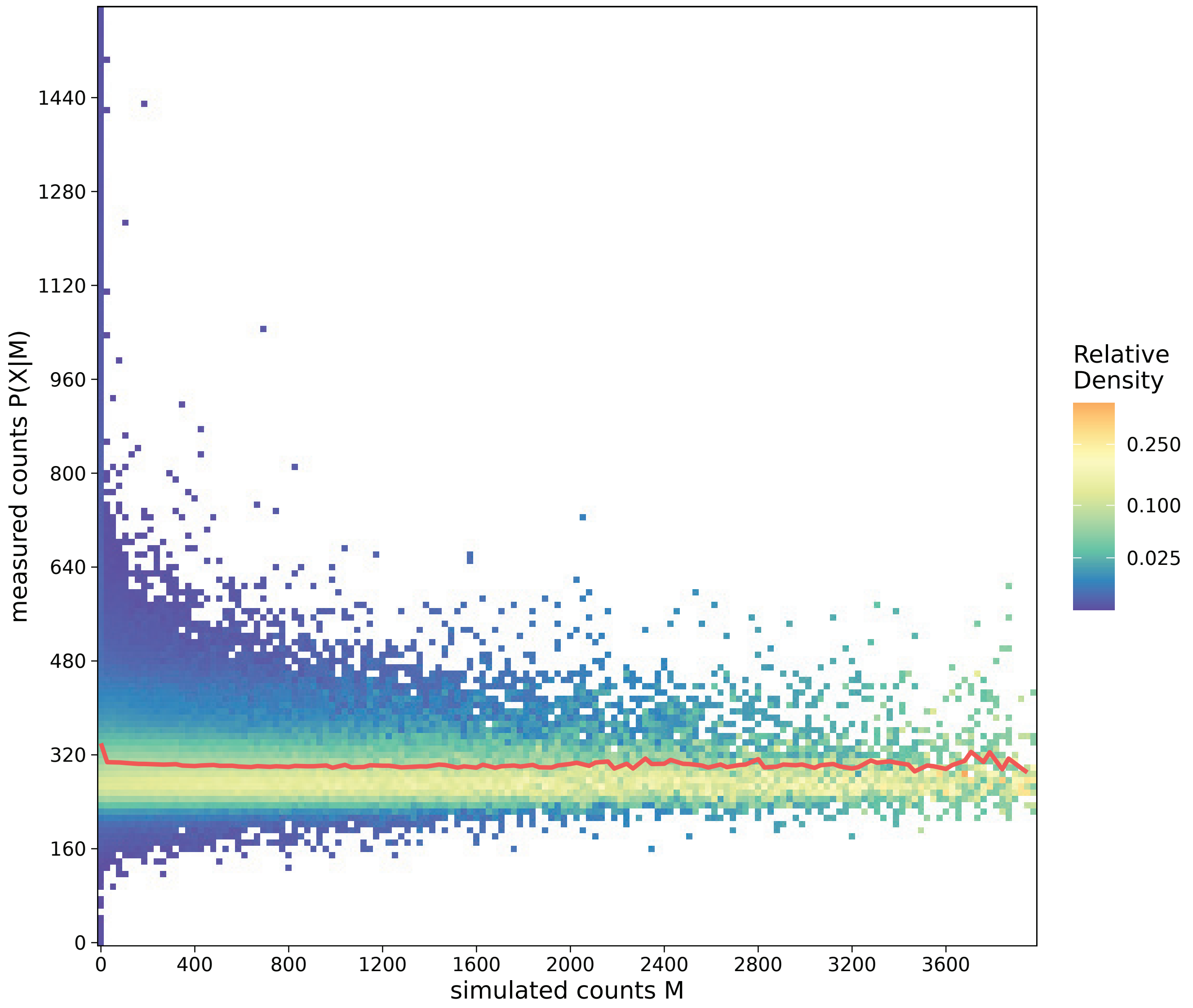

Figure S4 Alu mappability analysis — To check if mappability has a systematic effect on our measured Alu expression, we calculated a mappability score for each Alu element.

We simulated a homogenious coverage of paired-end Illumina reads using pIRS v2.0.0 (Hu 2012) with the same sequencing characteristics as those of our real sequencing experiment (see Methods). We then mapped those simulated reads back to the genome using the STAR aligner with the same settings as those used in our real sequencing experiment (see Methods and Schwalb 2016), and calculated the length corrected read counts for each Alu loci.

If there was a bias caused by mappability, the measured Alu expression would depend on this mappability. In other words, the distribution of Alu expression X, given a certain mappability score M, P(X|M), should vary. In particular, its expectation value, E(P(X|M)), should increase with increasing mappability.

The heatmap shows P(X|M) as a function of M. Each plotted column corresponds to one such distribution P(X|M) for one value of M. Relative density is color coded following the palette given on the right. The expectation value E(X|M) is indicated by the red line for each M. The figure demonstrates, that there is no systematic variation of E(X|M) with M, and that P(X|M) does not vary substantially. Thus, there is no bias caused by Alu mappability, which is also corroborated by the findings of Sexton *et al.* (2019), who show that the mappability of transposable elements can be improved through the use of paired-end read libraries to the point where a majority of elements are uniquely mappable.
