# Supplementary material for: RNA transcription and degradation of Alu retrotransposons depends on sequence features and evolutionary history
Source: G3 (Bethesda). 2022 Mar 7;12(5):jkac054. doi: 10.1093/g3journal/jkac054 (PMC9073682; doi:10.1093/g3journal/jkac054)
Supplement: jkac054_Supplement_S5 [file jkac054_supplement_s5.pdf]

Explanation of how we come to model the degradation rate δ^1 of Alu element transcripts.

In our experiment, we measured Alu element expression using DTA (dynamic transcriptome analysis) with 4sU labeling. In this protocol, newly created transcripts are labeled, making them distinguishable from transcripts created before the labeling pulse.
In the end, we want to obtain an estimate of the half-life $t_{1/2}$ of each individual Alu element, which can be calculate from the degradation rate δ by

$$t_{1/2} = \frac{\ln(2)}{\delta}$$

The labeling pulse's duration Δt is 5 min, meaning that 5 min passed after the labeling agent was added and before the amount of labeled transcripts within the cell was measured.

With time, new transcripts are created and old transcript are degraded. This means that after the labeling pulse, the amount of unlabeled transcripts continually decreases, because all transcripts created after the labeling pulse are labeled, until only labeled transcripts remain. We assume that transcript degradation follows exponential decay, while the total amounts of transcripts in a cell remains constant, as transcription and transcript decay are in equilibrium. Therefore, the total amount of transcripts remains constant, but the ratio of labeled reads increases exponentially.

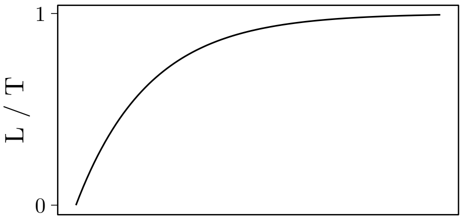

Let t_a be the total number of molecules of any Alu element a in solution. Among those t_a molecules, let l_a denote the number of newly synthesized (and therefore labeled) molecules. By assumption of steady state conditions and an exponential decay, the ratio between labeled and total molecules r_a for any Alu element a is given by

$$r_a = {}^{l_a}/_{t_a} = 1 - \exp(-\delta_a \Delta t)$$
$$\ln r_a = \ln(1 - \exp(-\delta_a \Delta t))$$

where δ_a is the degradation rate of any Alu element a. The total amount of molecules t_a of any Alu element a is also given as:

$$t_a = {}^{\mu_a}/_{\delta_a}$$

where μ_a is the synthesis rate of any Alu element a.
Neglecting the decay of newly synthesized transcripts, we likewise obtain[2]

$$l_a = \mu_a \Delta t$$

---

1 Usually, the degradation rate is given the symbol λ, but as λ is already in use as a parameter of the Poisson distribution, δ is used instead.

2 This formula is an approximation. It might be replaced by the solution of the standard ODE for RNA metabolism. Or, even more difficult, it might be replaced by a term that takes into account the non-constant labeling efficiency for short labeling periods.

We further assume that

$$l_{all} = \sum_a l_a \quad \text{and} \quad t_{all} = \sum_a t_a$$

We now shift our view from the molecules in solution to read counts obtained through sequencing. We prepare a sequencing library with N_{tot} reads. After 4sU pull-down, we prepare a sequencing library of size N_{lab} reads. The distribution of total counts T_a and labeled counts L_a respectively for any Alu element a is then[3]

$$T_a \sim \text{Pois}(\lambda_{tot} = \frac{t_a}{t_{all}} \cdot N_{tot})$$
$$L_a \sim \text{Pois}(\lambda_{lab} = \frac{l_a}{l_{all}} \cdot N_{lab})$$

We thus assume that the <u>estimated</u> ratio between labeled and total molecules $\hat{r}_a$ for any Alu element a is given by

$$\hat{r}_a = \frac{L_a}{T_a} \cdot \frac{N_{tot}}{N_{lab}}$$

We still need to estimate l_{all}/t_{all} i.e., the fraction of (all) labeled molecules among all molecules in solution. We use spike-ins to do so. Let l_{spk} be the number of labeled spike-in molecules, and t_{spk} the number of total spike-in molecules that were added to the solution. We know that

$$\frac{l_{spk}}{l_{all}} \approx \frac{L_{spk}}{L_{all}} = \frac{L_{spk}}{N_{lab}} \quad \text{and} \quad \frac{t_{spk}}{t_{all}} \approx \frac{T_{spk}}{T_{all}} = \frac{T_{spk}}{N_{tot}} \tag{1}$$

The term $q = l_{spk}/t_{spk}$ is thus known by composition of the spike-in reagents.

We use maximum likelihood estimation and the trick

$$r_a = \frac{l_a}{t_a} = \frac{l_a}{t_a} \cdot \frac{l_{spk}}{l_{spk}} \cdot \frac{t_{spk}}{t_{spk}}$$

as l_{spk}/l_{spk} and t_{spk}/t_{spk} are both 1, but can be rearranged to

$$= \frac{l_a}{l_{spk}} \cdot \frac{t_{spk}}{t_a} \cdot \frac{l_{spk}}{t_{spk}}$$
$$= \frac{l_a}{l_{spk}} \cdot \frac{t_{spk}}{t_a} \cdot q \tag{2}$$

We now assume that the ratio between spike-in molecules and Alu-specific molecules in solution for both fractions is approximately preserved during sequencing

$$\frac{l_a}{l_{spk}} \approx \frac{L_a}{L_{spk}} \quad \text{and} \quad \frac{t_{spk}}{t_a} \approx \frac{T_{spk}}{T_a} \tag{3}$$

Solving equation 2 suitably, we can obtain representations of both t_a/t_{all} and l_a/l_{all} free from any "in-solution" quantities (denoted by lower case variables), using only known "read-count"

[3] There is also the possibility to choose a (zero-inflated) negative binomial distribution instead.

quantities (denoted by upper case variables), except for q, which is also known, and r_a of course, which we want to estimate.

$$
\begin{aligned}
r_a &\overset{2}{=} \frac{l_a}{l_{spk}} \cdot \frac{t_{spk}}{t_a} \cdot q \\
\frac{t_a}{t_{all}} \cdot r_a &= \frac{\cancel{t_a}}{t_{all}} \cdot \frac{l_a}{l_{spk}} \cdot \frac{t_{spk}}{\cancel{t_a}} \cdot q \\
\frac{t_a}{t_{all}} &= \frac{l_a}{l_{spk}} \cdot \frac{t_{spk}}{t_{all}} \cdot \frac{1}{r_a} \cdot q \\
\frac{t_a}{t_{all}} &\overset{1,3}{\approx} \frac{L_a}{L_{spk}} \cdot \frac{T_{spk}}{N_{tot}} \cdot \frac{1}{r_a} \cdot q
\end{aligned}
\tag{4}
$$

For l_a/l_{all}, we begin with this tautology, which we multiply by $r_a \;/\; (l_a/l_{spk} \cdot t_{spk}/t_a \cdot q)$, as this is 1 according to (2).

$$
\begin{aligned}
\frac{l_a}{l_{all}} &= \frac{l_a}{l_{all}} \\
&= \frac{l_a}{l_{all}} \cdot \left(\frac{r_a}{\frac{l_a}{l_{spk}} \cdot \frac{t_{spk}}{t_a} \cdot q} \right) \\
&= \frac{l_{spk}}{l_{all}} \cdot \frac{t_a}{t_{spk}} \cdot r_a \cdot \frac{1}{q} \\
&\overset{1,3}{\approx} \frac{L_{spk}}{N_{lab}} \cdot \frac{T_a}{T_{spk}} \cdot r_a \cdot \frac{1}{q}
\end{aligned}
\tag{5}
$$

Using 4 and 5, we write the expected occurrence rate for total reads λ_{tot} and labeled reads λ_{lab}

$$
\begin{aligned}
\lambda_{tot} &= N_{tot} \cdot \frac{t_a}{t_{all}} \\
&\overset{4}{=} N_{tot} \cdot \frac{L_a}{L_{spk}} \cdot \frac{T_{spk}}{N_{tot}} \cdot \frac{1}{r_a} \cdot q \\
&= \underbrace{\frac{L_a}{L_{spk}} \cdot T_{spk} \cdot q}_{=c_{tot}} \cdot r_a^{-1} = \boxed{c_{tot} \cdot r_a^{-1}}
\end{aligned}
\tag{6}
$$

$$
\begin{aligned}
\lambda_{lab} &= N_{lab} \cdot \frac{l_a}{l_{all}} \\
&\overset{5}{=} N_{lab} \cdot \frac{L_{spk}}{N_{lab}} \cdot \frac{T_a}{T_{spk}} \cdot r_a \cdot \frac{1}{q} \\
&= \underbrace{L_{spk} \cdot \frac{T_a}{T_{spk}} \cdot \frac{1}{q}}_{=c_{lab}} \cdot r_a = \boxed{c_{lab} \cdot r_a}
\end{aligned}
\tag{7}
$$

We can now write down the likelihood function $\mathcal{L}$ of observing a specific T_a and L_a, as a parameterized combination of the individual two Poisson probability mass functions, which are given by

$$
\mathrm{P}(k; \lambda) = \frac{\lambda^k \cdot \mathrm{e}^{-k}}{k!} = \mathrm{e}^{-\lambda} \cdot \frac{\lambda^k}{k!}
$$

where k is number of occurrences. In our case, this is T_a and L_a respectively.

$$\begin{aligned}
\mathcal{L}(L_a, T_a; r_a, q, L_{spk}, T_{spk}) &= \mathrm{Pois}(T_a; \lambda_{tot} = c_{tot} \cdot r_a^{-1}) \cdot \mathrm{Pois}(L_a; \lambda_{lab} = c_{lab} \cdot r_a^{-1}) \\
&= \mathrm{e}^{-\lambda_{tot}} \frac{\lambda_{tot}^{T_a}}{T_a!} \cdot \mathrm{e}^{-\lambda_{lab}} \frac{\lambda_{lab}^{L_a}}{L_a!} \\
&= \mathrm{e}^{-\left(c_{tot} \cdot r_a^{-1}\right)} \frac{\left(c_{tot} \cdot r_a^{-1}\right)^{T_a}}{T_a!} \cdot \mathrm{e}^{-\left(c_{lab} \cdot r_a\right)} \frac{\left(c_{lab} \cdot r_a\right)^{L_a}}{L_a!}
\end{aligned}$$

We now ignore all constants without direct influence on r_a, as they do not influence the maximization of $\mathcal{L}$ with regards to r_a.

$$\begin{aligned}
&\propto \mathrm{e}^{-\left(c_{tot} \cdot r_a^{-1}\right)} \cdot r_a^{-T_a} \cdot \mathrm{e}^{-\left(c_{lab} \cdot r_a\right)} \cdot r_a^{L_a} \\
&= \exp\left(-\frac{c_{tot}}{r_a} - c_{lab} \cdot r_a\right) \cdot r_a^{L_a - T_a}
\end{aligned}$$

To find the optimal r_a, and thereby the degradation rate δ_a and the half-life $t_{1/2}$, for any Alu element a, we maximize the log likelihood function ℓ

$$\begin{aligned}
\ell(r_a; L_a, T_a, q, L_{spk}, T_{spk}) &= -\frac{c_{tot}}{r_a} - c_{lab} \cdot r_a + (L_a - T_a) \cdot \ln(r_a) \\
&\overset{6,7}{=} -\frac{\left(L_a/L_{spk} \cdot T_{spk} \cdot q\right)}{r_a} - \left(L_{spk} \cdot \frac{T_a}{T_{spk}} \cdot \frac{1}{q}\right) \cdot r_a + (L_a - T_a) \cdot \ln(r_a) \\
&= L_a \left(\ln(r_a) - \frac{T_{spk}}{L_{spk}} \frac{q}{r_a}\right) + T_a \left(-\frac{L_{spk}}{T_{spk}} \frac{r_a}{q} - \ln(r_a)\right)
\end{aligned}$$

Assuming the log likelihood function is well-behaved, the maximum of ℓ lies where the partial derivative of ℓ with respect to r_a is 0.

$$\frac{\partial \ell}{\partial r_a} = \frac{L_a \cdot (L_{spk} \cdot r_a + T_{spk} \cdot q)}{L_{spk} \cdot r_a^2} - \frac{T_a \cdot (L_{spk} \cdot r_a + T_{spk} \cdot q)}{T_{spk} \cdot r_a \cdot q} := 0$$

Under the valid assumtion that r_a, L_a, T_a, q, L_{spk}, and T_{spk} are all positive, this collapses to

$$r_a = \frac{L_a}{T_a} \cdot \frac{T_{spk}}{L_{spk}} \cdot q$$

■
