# Supplementary material for: RNA transcription and degradation of Alu retrotransposons depends on sequence features and evolutionary history
Source: G3 (Bethesda). 2022 Mar 7;12(5):jkac054. doi: 10.1093/g3journal/jkac054 (PMC9073682; doi:10.1093/g3journal/jkac054)
Supplement: jkac054_Supplement_S6 [file jkac054_supplement_s6.pdf]

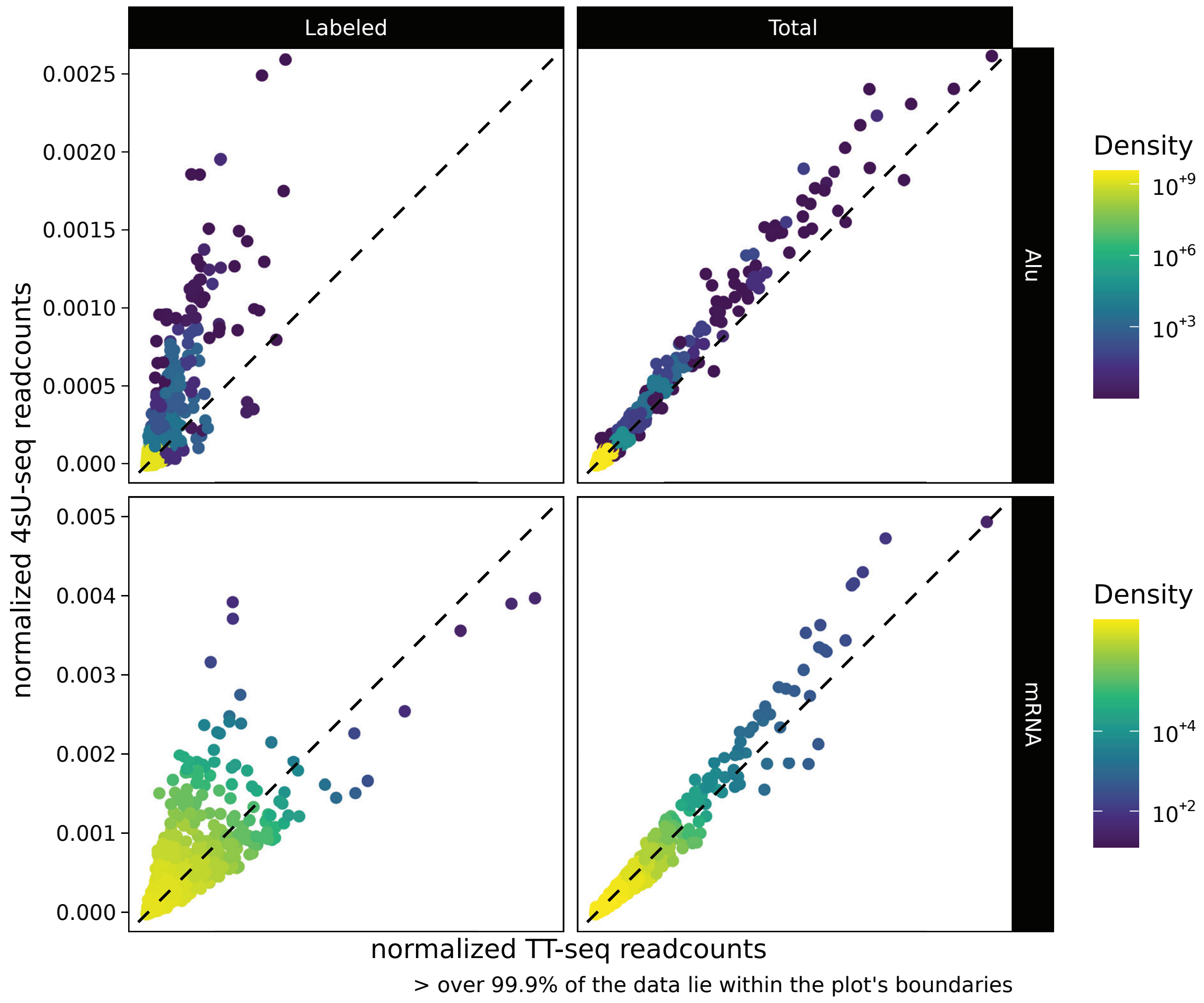

Figure S6 Correlation between sequencing methods — Scatterplot showing the correlation between the sequencing methods TT-seq (horizontal axis) and 4sU-seq (vertical axis) used for the half-life estimation via MLE. Shown are readcounts rescaled to the fraction of total reads for each group with merged replicates, excluding values of 0. The color represents point density (shown right). Both sequencing methods were used for the estimation, as a Spearman correlation r > 0.80 was observed between TT-seq and 4sU-seq readcounts in all groups. Replicates were merged, as they also showed correlation of r > 0.80 in all comparisons.
