# Supplementary material for: RNA transcription and degradation of Alu retrotransposons depends on sequence features and evolutionary history
Source: G3 (Bethesda). 2022 Mar 7;12(5):jkac054. doi: 10.1093/g3journal/jkac054 (PMC9073682; doi:10.1093/g3journal/jkac054)
Supplement: jkac054_Supplement_S7 [file jkac054_supplement_s7.pdf]

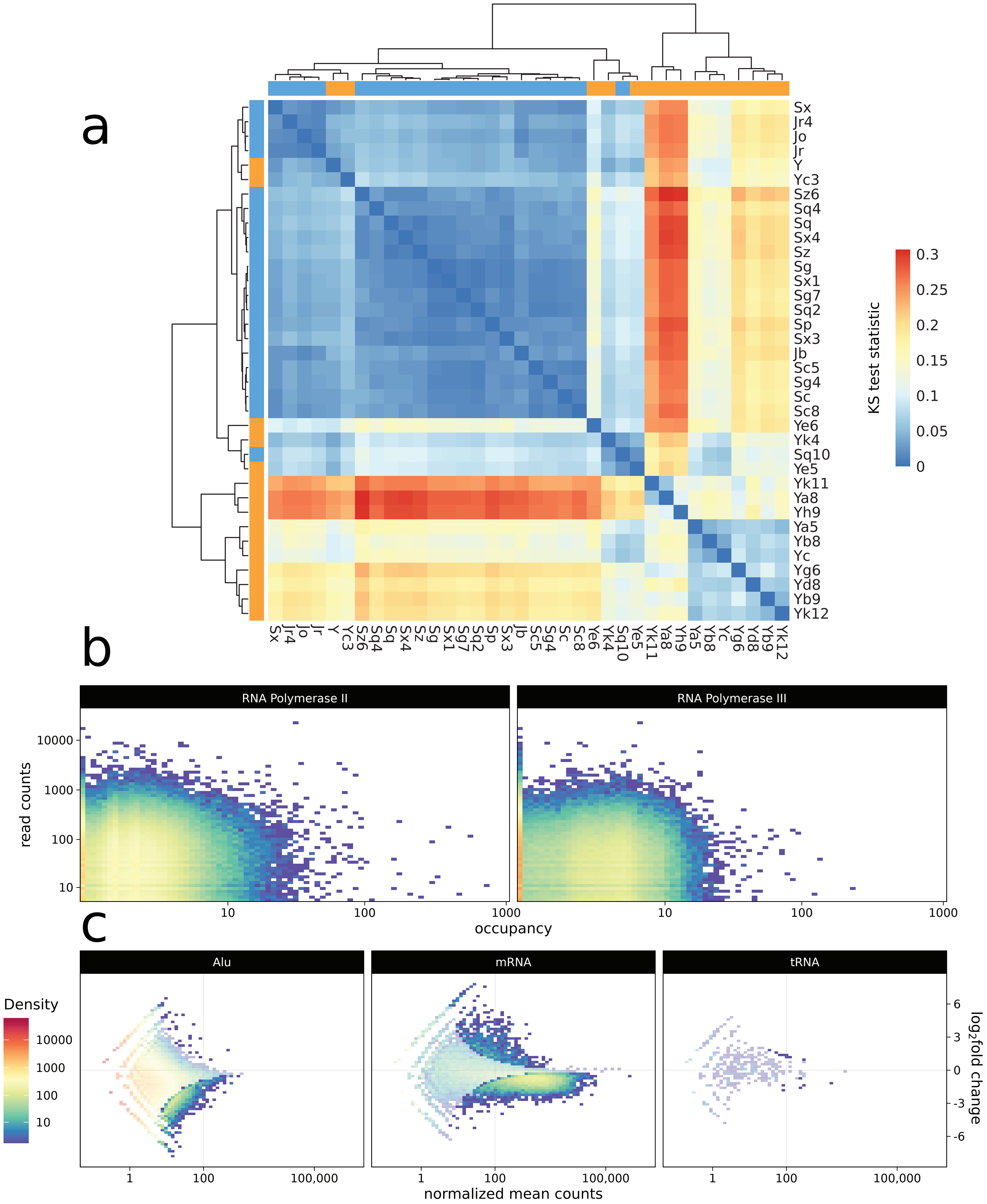

Figure S7 Supplemental figures. **a**) Heatmap of the pairwise KS test statistic between all Alu families with hierarchical clustering applied. AluY families are clearly separated from the older families, with the exception of AluSq10 and AluYc3 (AluYc2), as indicated by the colored bar (blue indicating old and yellow indicated young families). **b**) 2D density heatmap showing the lack of correlation between Alu element read counts and Pol-II (left) and Pol-III (right) occupancy. **c**) 2D density heatmap showing the DESeq2 differential expression of Alu elements, mRNAs, and tRNAs as control under a-amanitin Pol-II inhibition, using spike-in control as normalization instead of mtRNAs. Semi-transparent areas do not pass the significance threshold. Both Alu elements and mRNAs show stronger significant down- than upregulation.
