# Supplementary material for: RNA transcription and degradation of Alu retrotransposons depends on sequence features and evolutionary history
Source: G3 (Bethesda). 2022 Mar 7;12(5):jkac054. doi: 10.1093/g3journal/jkac054 (PMC9073682; doi:10.1093/g3journal/jkac054)
Supplement: jkac054_Supplement_S9 [file jkac054_supplement_s9.pdf]

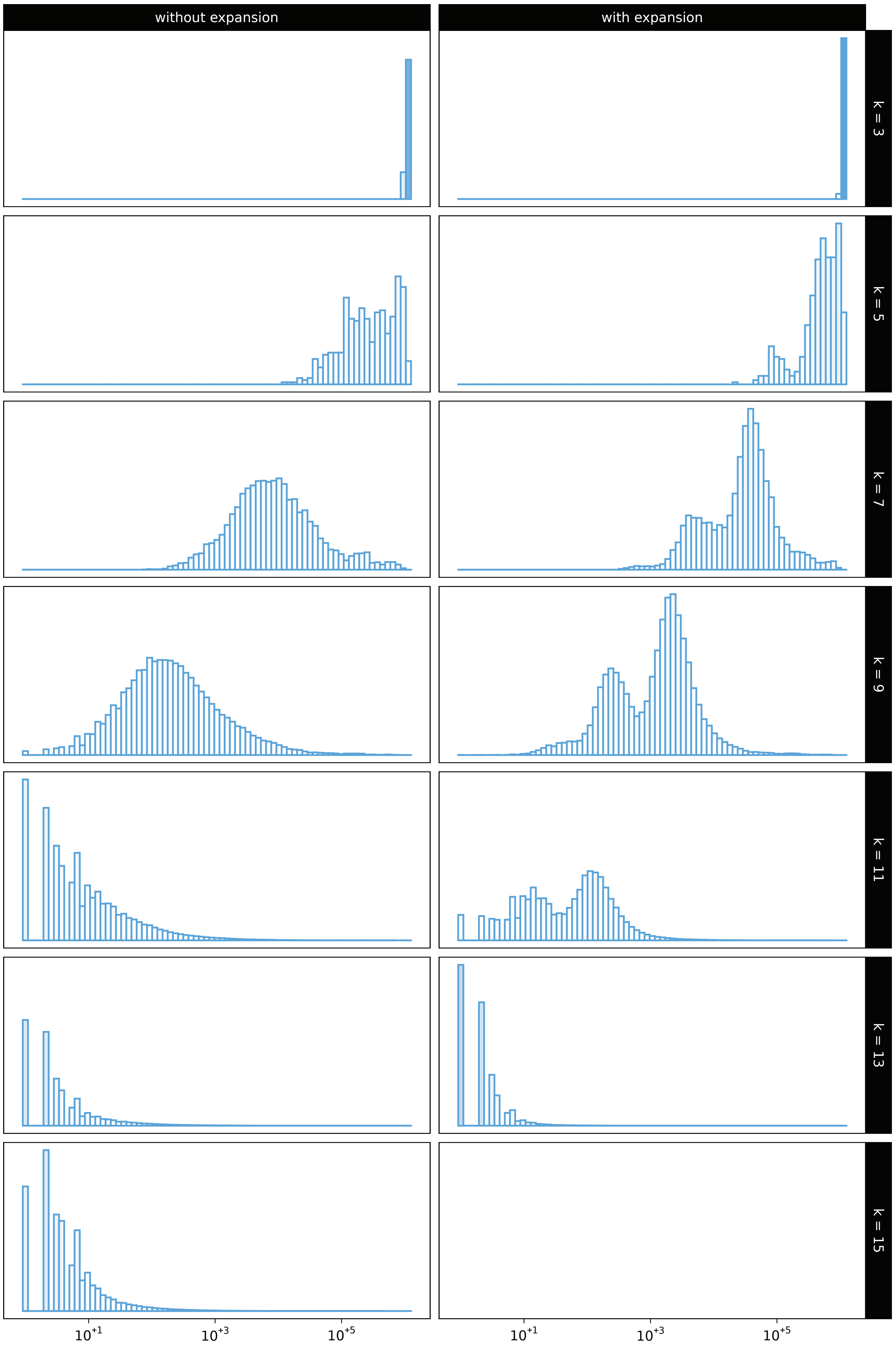

Figure S9 Choice of k — Histogram of the Alu sequences associated with each individual node in the de Bruijn graph depending on the choice of k and whether or not to expand the Alu sequences by 100 bp up- and downstream. k = 9 results in a smooth distribution of sequences per node for both cases. The bimodal distribution observable when including 100 bp up- and downstream of the Alu sequences results from the differences in sequence variability between the Alu sequence itself and the flanking regions.
