# Supplementary material for: RNA transcription and degradation of Alu retrotransposons depends on sequence features and evolutionary history
Source: G3 (Bethesda). 2022 Mar 7;12(5):jkac054. doi: 10.1093/g3journal/jkac054 (PMC9073682; doi:10.1093/g3journal/jkac054)
Supplement: jkac054_Supplement_S10 [file jkac054_supplement_s10.pdf]

Table S10 Detailed results of the JASPAR database search (Fornes 2020, relative profile score threshold of 80%) as shown in Table 1. In addition to the information reproduced directly from the JASPAR database, the column *Pol-II Reference* lists references for the Pol-II specificity of each matching transcription factor. NTNU SB: inferred from NTNU SB sequence alignment according to the UniProt database (Bateman 2021). Ensemble: Extracted from the Ensemble database (Howe 2021). BHF-UCL: Extracted from the BHF-UCL gene association of the Cardiovascular Gene Ontology Annotation Initiative (Zheng 2011).

	k-mer	OR	JASPAR match	Matrix ID	Score	Relative Score	Start	End	Strand	Predicted Sequence	Pol-II Reference
1	AACGCGCCA	2.65	—								
2	ATCGCCCGC	2.72	NFIX	MA0671.1	3.2396	0.8217	1	9	+	ATCGCCCGC	NTNU SB
			NR2C2 (var.2)	MA1536.1	0.2758	0.8182	1	8	-	GGCGTTGA	NTNU SB
3	CGGACTGCT	2.07	MEIS1	MA0498.2	1.0797	0.8036	1	7	+	CGGACTG	Gaudet 2011
			TEAD3	MA0808.1	2.5206	0.8314	1	8	-	GCAGTCCG	NTNU SB
4	CTCAACGCC	2.40	SOX18	MA1563.1	7.2890	0.9187	1	8	+	CTCAACGC	Hoeth 2012
			BARHL1	MA0877.2	4.5846	0.8548	1	8	+	CTCAACGC	NTNU SB
			ZNF354C	MA0130.1	4.6356	0.8126	1	6	+	CTCAAC	Gaudet 2011
			NR2C2 (var.2)	MA1536.1	0.5668	0.8235	1	8	-	GCGTTGAG	NTNU SB
			GSX2	MA0893.2	2.0604	0.8152	2	9	-	GGCGTTGA	DeMori 2019
5	GAAACCGTC	2.12	—								
6	GACACGCGC	2.27	ARNT::HIF1A	MA0259.1	7.7207	0.8962	2	9	-	GCGCGTGT	Huang 2009
			TFE3	MA0831.2	6.7330	0.8555	1	8	-	CGCGTGTC	Ensembl
			USF1	MA0093.1	5.7024	0.8008	2	8	-	CGCGTGT	BHF-UCL
7	GATCGCCCG	2.56	GATA2	MA0036.1	4.6275	0.8825	1	5	-	CGATC	Ensembl
8	GGCGGACTG	2.35	MEIS1	MA0498.2	1.0797	0.8036	3	9	+	CGGACTG	Gaudet 2011
9	GGGCGGACT	2.64	—								
10	TAGGCGCGC	2.08	—								
11	TCAACGCCT	2.22	TBX4	MA0806.1	5.6304	0.8382	2	9	-	AGGCGTTG	Yi 2000
			TBX5	MA0807.1	5.8659	0.8193	2	9	-	AGGCGTTG	BHF-UCL
			NR2C2 (var.2)	MA1536.1	0.2758	0.8182	1	8	-	GGCGTTGA	NTNU SB
			GSX2	MA0893.2	2.0604	0.8152	1	8	-	GGCGTTGA	DeMori 2019
			MGA	MA0801.1	3.8722	0.8126	2	9	-	AGGCGTTG	NTNU SB
12	TGACACGCG	2.92	FOS::JUN	MA0099.2	5.7234	0.8196	1	7	+	TGACACG	Kodeboyina 2010
			TFE2	MA0831.2	6.7330	0.8555	2	9	-	CGCGTGTC	BHF-UCL
			TBX4	MA0806.1	5.9236	0.8441	1	8	-	GCGTGTCA	Yi 2000
			MGA	MA0801.1	5.3019	0.8386	1	8	-	GCGTGTCA	NTNU SB
			TBX5	MA0807.1	6.0479	0.8240	1	8	-	GCGTGTCA	BHF-UCL
			USF1	MA0093.1	5.7024	0.8008	3	9	-	CGCGTGT	BHF-UCL

Bateman 2021 Bateman, A., Martin, M. J., Orchard, S., Magrane, M., Agivetova, R., Ahmad, S., Alpi, E., Bowler-Barnett, E. H., Britto, R., Bursteinas, B., Bye-A-Jee, H., Coetzee, R., Cukura, A., Silva, A. Da, Denny, P., Dogan, T., Ebenezer, T. G., Fan, J., Castro, L. G., … Zhang, J. (2021). *UniProt: The universal protein knowledgebase in 2021.* Nucleic Acids Research, 49(D1), D480–D489. https://doi.org/10.1093/nar/gkaa1100

Howe 2021 Howe, K. L., Achuthan, P., Allen, J., Allen, J., Alvarez-Jarreta, J., Amode, M. R., Armean, I. M., Azov, A. G., Bennett, R., Bhai, J., Billis, K., Boddu, S., Charkhchi, M., Cummins, C., Da Rin Fioretto, L., Davidson, C., Dodiya, K., El Houdaigui, B., Fatima, R., … Flicek, P. (2021). *Ensembl 2021.* Nucleic Acids Research, 49(D1), D884–D891. https://doi.org/10.1093/nar/gkaa942

Fornes 2020 Fornes, O., Castro-Mondragon, J. A., Khan, A., Van Der Lee, R., Zhang, X., Richmond, P. A., Modi, B. P., Correard, S., Gheorghe, M., Baranašić, D., Santana-Garcia, W., Tan, G., Chèneby, J., Ballester, B., Parcy, F., Sandelin, A., Lenhard, B., Wasserman, W. W., & Mathelier, A. (2020). *JASPAR 2020: Update of the open-Access database of transcription factor binding profiles.* Nucleic Acids Research, 48(D1), D87–D92. https://doi.org/10.1093/nar/gkz1001

DeMori 2019 De Mori, R., Severino, M., Mancardi, M. M., Anello, D., Tardivo, S., Biagini, T., Capra, V., Casella, A., Cereda, C., Copeland, B. R., Gagliardi, S., Gamucci, A., Ginevrino, M., Illi, B., Lorefice, E., Musaev, D., Stanley, V., Micalizzi, A., Gleeson, J. G., … Valente, E. M. (2019). *Agenesis of the putamen and globus pallidus caused by recessive mutations in the homeobox gene GSX2.* Brain, 142(10), 2965–2978. https://doi.org/10.1093/brain/awz247

Hoeth 2012 Hoeth, M., Niederleithner, H., Hofer-Warbinek, R., Bilban, M., Mayer, H., Resch, U., Lemberger, C., Wagner, O., Hofer, E., Petzelbauer, P., & de Martin, R. (2012). *The Transcription Factor SOX18 Regulates the Expression of Matrix Metalloproteinase 7 and Guidance Molecules in Human Endothelial Cells.* PLOS ONE, 7(1), e30982. https://doi.org/10.1371/journal.pone.0030982

Gaudet 2011 Gaudet, P., Livstone, M. S., Lewis, S. E., & Thomas, P. D. (2011). *Phylogenetic-based propagation of functional annotations within the Gene Ontology consortium.* Briefings in Bioinformatics, 12(5), 449–462. https://doi.org/10.1093/bib/bbr042

Zheng 2011 Zheng, H., Wang, H., & Azuaje, F. (2011). *Incorporation of Ontology-driven biological knowledge into cardiovascular genomics.* 2011 Computing in Cardiology, 565–568.

Kodeboyina 2010 Kodeboyina, S., Balamurugan, P., Liu, L., & Pace, B. S. (2010). *cJun modulates Gγ-globin gene expression via an upstream cAMP response element.* Blood Cells, Molecules, and Diseases, 44(1), 7–15. https://doi.org/https://doi.org/10.1016/j.bcmd.2009.10.002

Huang 2009 Huang, X., Ding, L., Bennewith, K. L., Tong, R. T., Welford, S. M., Ang, K. K., Story, M., Le, Q.-T., & Giaccia, A. J. (2009). *Hypoxia-Inducible mir-210 Regulates Normoxic Gene Expression Involved in Tumor Initiation.* Molecular Cell, 35(6), 856–867. https://doi.org/10.1016/j.molcel.2009.09.006

Yi 2000 Yi, C. H., Russ, A., & Brook, J. D. (2000). *Virtual cloning and physical mapping of a human T-box gene, TBX4.* Genomics, 67(1), 92–95. https://doi.org/10.1006/geno.2000.6222
