# Supplementary material for: RNA transcription and degradation of Alu retrotransposons depends on sequence features and evolutionary history
Source: G3 (Bethesda). 2022 Mar 7;12(5):jkac054. doi: 10.1093/g3journal/jkac054 (PMC9073682; doi:10.1093/g3journal/jkac054)
Supplement: jkac054_Supplement_S11 [file jkac054_supplement_s11.pdf]

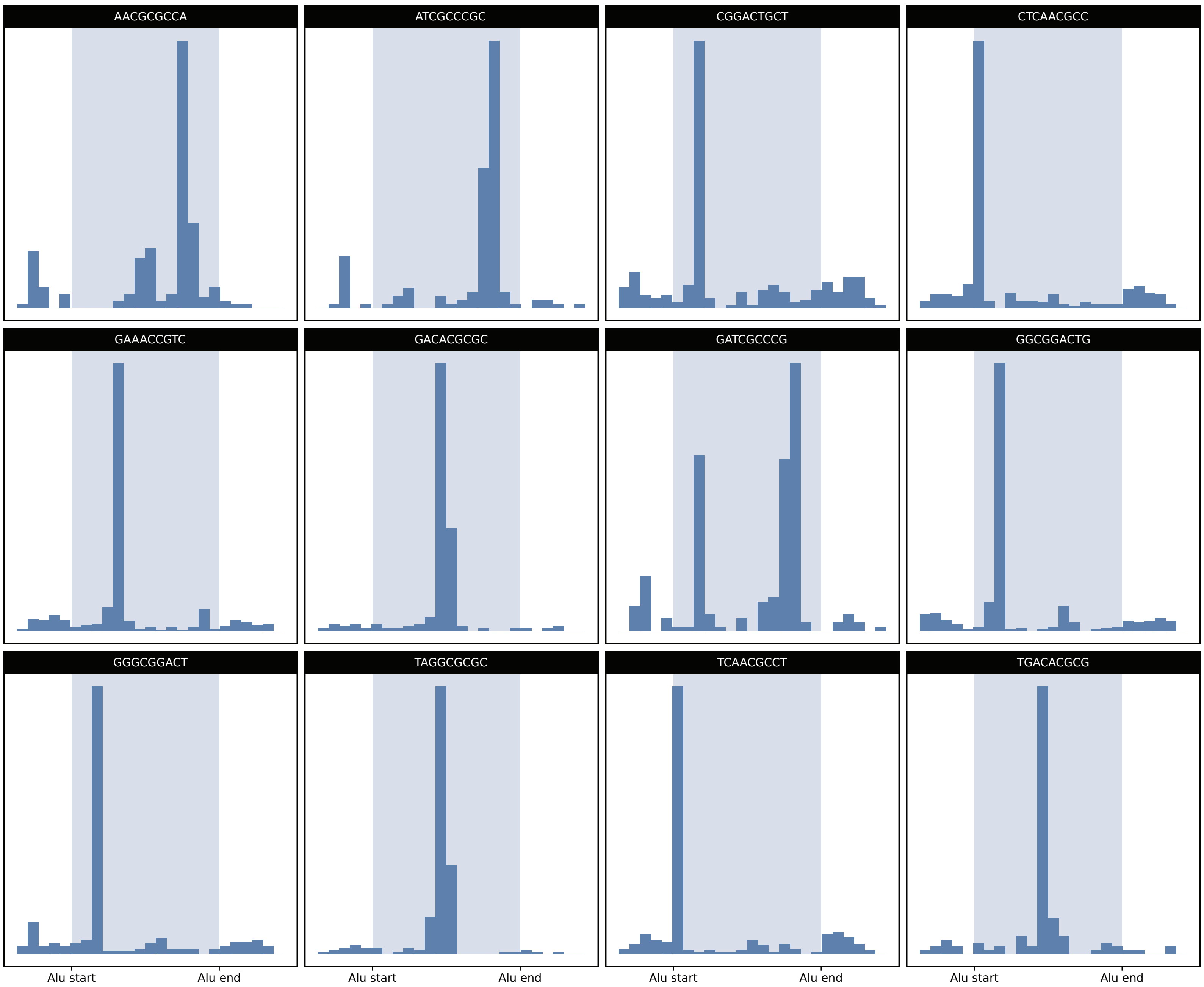

Figure S11 Relative Position of de Bruijn Graph k-mers — To make sure that the k-mers reported in Table 1 originate from the Alu sequence itself and not from the 100 bp flanking region that was included in the construction of the de Bruin graph (see Methods), we searched for each of the 12 k-mers in the sequences of all annotated Alu elements (UCSC Genome Browser).

Shown are histograms illustrating the number of k-mer sequence hits (y-axes: density) per binned relative location in all Alu elements (y-axes: relative location), relative because Alu elements vary slightly in their length. The blue area in each histogram denotes the actual Alu sequence, while the white area surrounding it left and right represents the 100 bp flanking region included in the graph construction.

For each of the 12 k-mers, the vast majority of search hits fall within the actual Alu sequence and not the flanking regions. The hits are also not situated in the variable A-rich region located in the center of the Alu sequence, but in the left or right arm. All k-mers exhibit one main location where they are found within the Alu sequence except for k-mer 7 GATCGCCCG (OR: 2.56, JASPAR hit: GATA2), which shows a secondary location of noteworthy accumulation.
